# Serum lipoprotein–derived fatty acids regulate hypoxia-inducible factor

**DOI:** 10.1074/jbc.RA120.015238

**Published:** 2021-01-13

**Authors:** Wei Shao, Jiwon Hwang, Chune Liu, Debaditya Mukhopadhyay, Shan Zhao, Meng-Chieh Shen, Ebru S. Selen, Michael J. Wolfgang, Steven A. Farber, Peter J. Espenshade

**Affiliations:** 1 Department of Cell Biology, Johns Hopkins University School of Medicine, Baltimore, Maryland, USA; 2 Department of Embryology, Carnegie Institution for Science, Baltimore, Maryland, USA; 3 Department of Biological Chemistry, Johns Hopkins University School of Medicine, Baltimore, Maryland, USA

**Keywords:** lipoprotein, low-density lipoprotein, fatty acid, lysosomal acid lipase, low-density lipoprotein (LDL), hypoxia-inducible factor (HIF), mitochondria

## Abstract

Oxygen regulates hypoxia-inducible factor (HIF) transcription factors to control cell metabolism, erythrogenesis, and angiogenesis. Whereas much has been elucidated about how oxygen regulates HIF, whether lipids affect HIF activity is un-known. Here, using cultured cells and two animal models, we demonstrate that lipoprotein-derived fatty acids are an independent regulator of HIF. Decreasing extracellular lipid supply inhibited HIF prolyl hydroxylation, leading to accumulation of the HIFα subunit of these heterodimeric transcription factors comparable with hypoxia with activation of downstream target genes. The addition of fatty acids to culture medium suppressed this signal, which required an intact mitochondrial respiratory chain. Mechanistically, fatty acids and oxygen are distinct signals integrated to control HIF activity. Finally, we observed lipid signaling to HIF and changes in target gene expression in developing zebrafish and adult mice, and this pathway operates in cancer cells from a range of tissues. This study identifies fatty acids as a physiological modulator of HIF, defining a mechanism for lipoprotein regulation that functions in parallel to oxygen.

Hypoxia-inducible factor (HIF) transcription factors are master regulators of oxygen homeostasis in humans, functioning in both normal physiology and disease (1, 2, 3). HIF transcription factors consist of an α subunit (HIFα) and a β subunit (HIFβ, also called ARNT), and oxygen controls HIF activity by regulating stability and transactivation function of the α subunit. In the presence of oxygen, prolyl hydroxylases (PHDs) modify two prolines, leading to ubiquitination of HIFα by the VHL E3 ligase and proteasomal degradation (4). PHD enzymes are Fe(II)– and 2-oxoglutarate–dependent oxygenases, whose activity requires oxygen, and consequently low oxygen inhibits PHD, leading to HIF activation (1). Under hypoxia, HIF activates expression of key genes regulating energy metabolism, erythrogenesis, and angiogenesis. HIF controls energy metabolism by up-regulating a glycolytic transcriptional program and diverting glycolytic products away from oxidation in the mitochondrion toward lactate production. This metabolic shift is a hallmark of cancer metabolism called the Warburg effect, making HIF an attractive target for anti-cancer therapy (5).

Cell growth requires a constant supply of lipids (6, 7), obtained either from the circulation or *de novo* synthesis. The membrane-bound sterol regulatory element–binding protein (SREBP) transcription factors are central regulators of lipid homeostasis (8) that respond to lipid availability to regulate both lipid uptake and synthesis. In mammals, two *SREBF* genes code for three SREBP proteins: SREBP-1a, SREBP-1c, and SREBP-2. In mouse liver where SREBP function is best studied, SREBP1 and SREBP2 control the supply of fatty acids and cholesterol, respectively (9). Newly synthesized SREBP proteins contain two transmembrane segments and bind to an escort protein SREBP cleavage–activating protein (SCAP) in the endoplasmic reticulum (ER). Under lipid-replete conditions, the SREBP-SCAP complex is retained in the ER by SCAP binding to insulin-induced gene (INSIG) proteins. ER-localized SREBPs are inactive because the N-terminal transcription factor remains bound to the membrane and cannot enter the nucleus. Under lipid-depleted conditions, the SREBP-SCAP complex dissociates from INSIG and traffics to the Golgi, where SREBP undergoes two sequential proteolytic cleavage events, releasing the N-terminal transcription factor domain from the membrane. SCAP functions as both an escort protein and a sterol sensor in this system and is essential for SREBP activity. In addition to SREBPs, evidence also indicates that HIF transcription factors act to maintain lipid supply as HIF stimulates lipid uptake (*FABP3/7*, *VLDLR*) (10, 11) and lipid storage (*PLIN2*, *ADFP*) (12, 13) and inhibits mitochondrial fatty acid oxidation (*MCAD*, *LCAD*) (14). Whether lipids also signal to HIF is unknown. Here, we demonstrate that lipoproteins regulate HIF activity in the presence of oxygen and provide evidence that serum lipoprotein–derived fatty acids are an independent regulator of HIF.

## Results

### Lipoprotein depletion activates HIFα under normoxia

**Figure 1:**
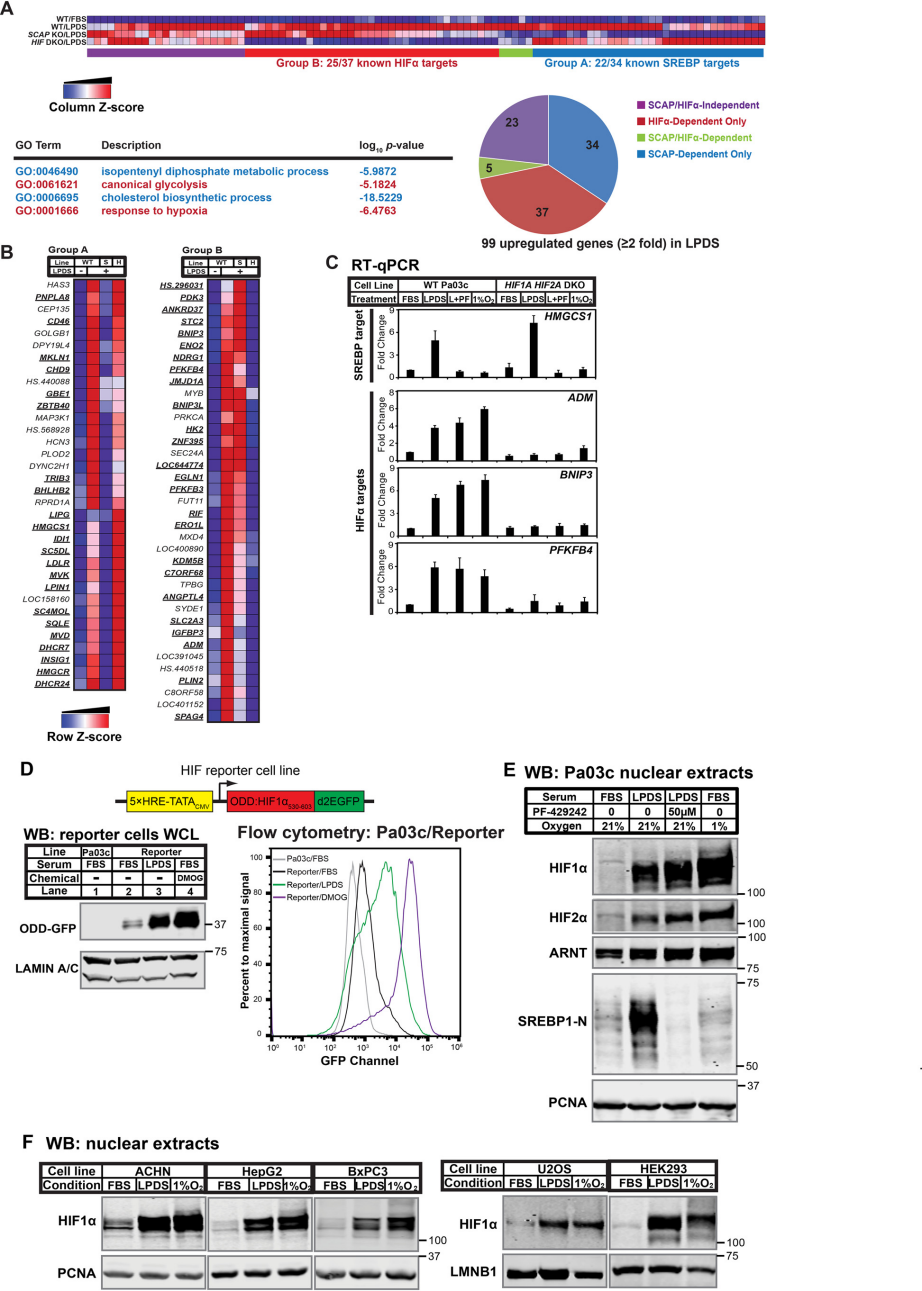
**Lipoprotein depletion activates HIFα under normoxia.***A*, patient-derived human PDAC cell line Pa03c (*WT*), *SCAP* KO cells (*S*), and *HIF1A HIF2A* DKO cells (*H*) were cultured in FBS or LPDS for 16 h. Gene expression was determined using Illumina bead arrays. LPDS-induced genes (≥2-fold) were analyzed for GO term enrichment using GOrilla/REVIGO. GO terms related to the SREBP or HIF pathway are *highlighted* in *blue* or *red*, respectively. A clustered heatmap of LPDS-induced genes was generated by GenePattern 2.0. *B*, a clustered heatmap of 99 genes induced upon lipoprotein depletion (≥2-fold) was generated by GenePattern. *Group A*, induction in LPDS required *SCAP*, but not *HIF*α. *Group B*, induction in LPDS required *HIF*α, but not *SCAP*. *Boldface*, *underlined genes* are known transcriptional targets of SREBP or HIF in Group A or B, respectively. *C*, WT or *HIF1A HIF2A* DKO Pa03c cells were cultured for 16 h in FBS in the presence of DMSO (0.1%) under normoxic (FBS) or hypoxic (1% O_2_) conditions or in LPDS in the presence of DMSO (0.1%) (LPDS) or Site-1 protease inhibitor PF-429242 (50 μm) (*L*+*PF*) to inhibit SREBP. Gene expression for SREBP or HIF transcriptional targets measured by RT-qPCR was normalized to vehicle-treated Pa03c cells cultured in FBS. *Error bars*, S.E. of -fold changes from three biological replicates (mean ± S.E.). *D*, diagram of HIF reporter cell line that is a Pa03c clone stably expressing HIF1α ODD-d2EGFP under the control of five tandem HREs. Shown are immunoblots (*WB*) of whole-cell lysates or flow cytometry analysis from parental Pa03c cells cultured for 24 h in FBS and HIF reporter cells cultured for 24 h in FBS, LPDS, or FBS with DMOG (1 mm), a cell-permeable prolyl-4-hydroxylase inhibitor. *E*, immunoblots of nuclear extracts from Pa03c cells cultured for 16 h in FBS in the presence of DMSO (0.1%) under normoxic or hypoxic (1% O_2_) conditions or in LPDS in the presence of DMSO (0.1%) or site-1 protease inhibitor PF-429242 (50 μm) to inhibit SREBP. PCNA served as a loading control. *F*, immunoblots of nuclear extracts from the indicated cell lines cultured for 16 h in FBS, LPDS, or FBS in 1% O_2_. PCNA or LMNB1 served as a loading control. *ACHN*, renal cell adenocarcinoma; *HepG2*, hepatocellular carcinoma; *BxPC3*, pancreatic adenocarcinoma; *U2OS*, osteosarcoma; *HEK293*, embryonic kidney.

Solid tumors are poorly vascularized, and how cancer cells regulate lipid homeostasis under these nutrient-depleted conditions is unknown. To investigate this, we probed the transcriptional response of patient-derived pancreatic adenocarcinoma Pa03c cells to low-lipid conditions by culturing cells in lipoprotein-deficient serum (LPDS). Removal of serum lipoproteins resulted in the induction (≥2-fold) of 99 genes. Gene ontology analysis of these 99 up-regulated genes indicated the activation of genes involved in mevalonate and cholesterol biosynthesis that are controlled by SREBP transcription factors (GO:0046490 and GO:0006695) (Fig. 1*A*). To test whether up-regulation of these genes was SREBP-dependent, we examined gene expression in Pa03c cells lacking the essential pathway component SCAP (9, 15). The induction of 34 of 99 genes required *SCAP* (Group A, Fig. 1, *A* and *B*). The majority of these genes were known SREBP targets, indicating that extracellular lipoproteins contributed to cellular lipid supply and negatively regulated SREBP transcription factors in pancreas cancer cells.

Unexpectedly, despite the fact that cells were cultured in the presence of oxygen, gene ontology analysis indicated that genes involved in glycolysis and the hypoxic response were also up-regulated (GO:0061621 and GO:0001666). Many of these up-regulated genes were known targets of HIF transcription factors (Group B, Fig. 1, *A* and *B*) (16, 17). Of the three HIFα proteins in higher metazoans, HIF1α and HIF2α (encoded by *HIF1A* and *HIF2A*) are transcriptional activators (1). We confirmed the HIF requirement for induction of these genes in LDPS using *HIF1A HIF2A* double knockout (DKO) Pa03c cells (Fig. 1 (*A–C*) and Fig. S1A). To monitor HIF activity in intact cells, we created a Pa03c HIF reporter cell line in which the transcription of *GFP* fused to the *HIF1A* oxygen-dependent degradation domain (ODD) is regulated by endogenous HIF activity and the degradation of ODD-GFP is controlled by the HIF PHDs (Fig. 1*D*). Consistent with the observed up-regulation of HIF target gene expression, immunoblotting, indirect immunofluorescence, and flow cytometry using the HIF reporter cell line demonstrated that lipoprotein removal activated HIF to levels comparable with 1% oxygen or treatment with *N*-(2-methoxy-2-oxoacetyl)glycine methyl ester (DMOG), a cell-permeable prolyl-4-hydroxylase inhibitor (Fig. 1 (*D* and *E*) and Fig. S1B). Both HIF1α and HIF2α increased in LPDS (Fig. 1*E*). As expected, lipoprotein depletion also activated SREBP-1 (Fig. 1*E*). Importantly, HIFα accumulation was independent of SREBP activity because treatment with the site-1 protease inhibitor PF-429242 blocked SREBP-1 cleavage but had no effect on HIFα induction or target gene expression (Fig. 1, *C* and *E*). Similarly, SREBP-1 activation did not require HIF, insomuch as SREBP target genes were induced in *HIF1A HIF2A* DKO cells (Fig. 1, *A–C*). Finally, this response was not restricted to a single cell line or cancer type as HIFα accumulated in LPDS to levels comparable with 1% oxygen in another five cell lines tested from different tumor types (Fig. 1*F*). Collectively, these data demonstrate that serum lipoprotein depletion activates HIFα under normoxia in multiple cancer cell lines.

### Low-density lipoproteins regulate HIFα

**Figure 2:**
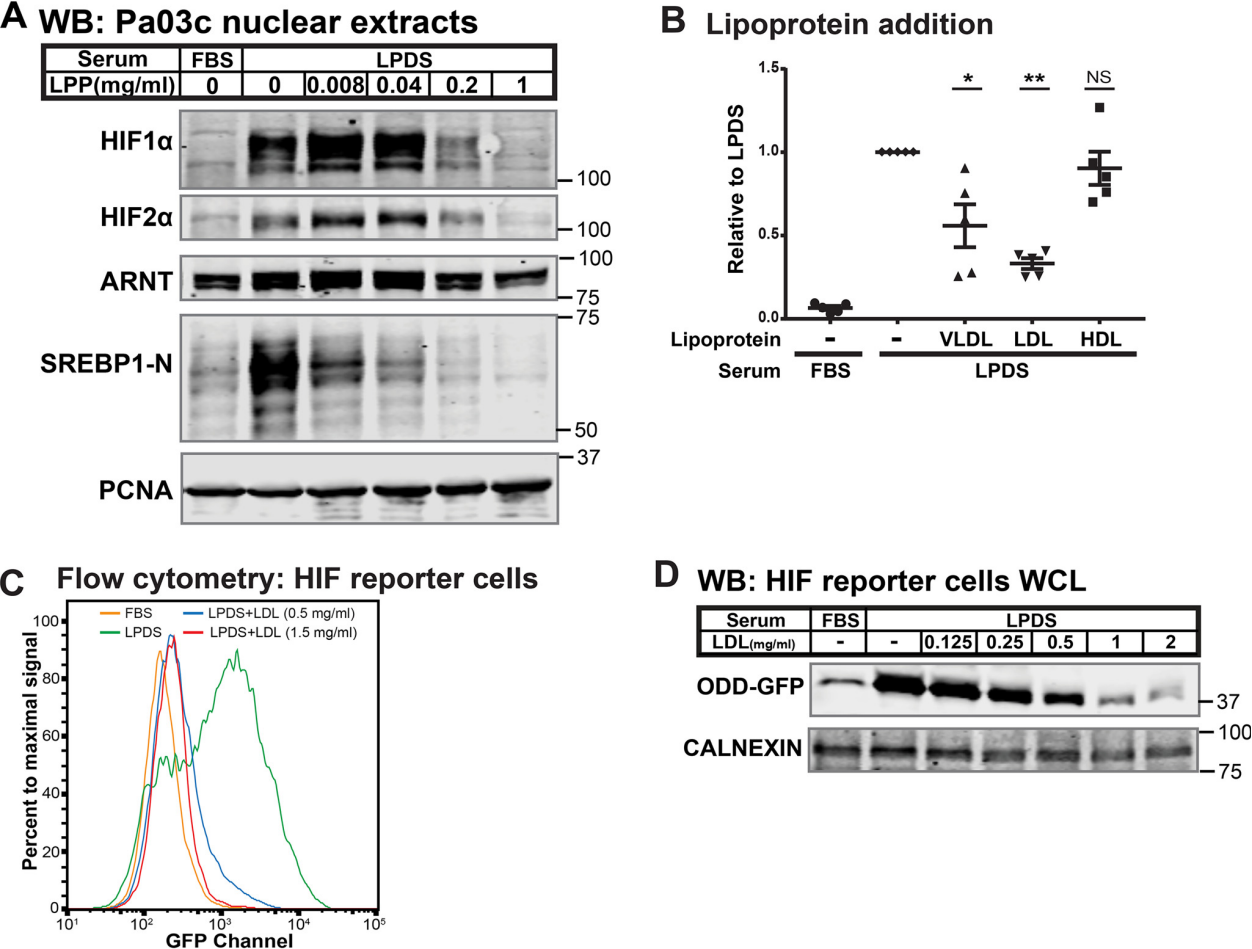
**Low-density lipoprotein regulates HIFα.***A*, immunoblots (*WB*) of nuclear extracts from Pa03c cells cultured for 16 h in FBS or LPDS supplemented with bovine lipoproteins (*LPP*; 0–1 mg/ml). PCNA served as a loading control. *B*, Pa03c cells were cultured for 16 h in FBS or LPDS with the indicated lipoprotein additions: human VLDL (0.2 mg/ml), human LDL (1 mg/ml), and human HDL (0.5 mg/ml). HIF1α immunoblot signal was normalized first to the loading control HDAC1 and then normalized to that in LPDS (*n* = 5, mean ± S.E. (*error bars*)). *p* values from a single-column *t* test (LPDS *versus* LPDS + VLDL, LDL, or HDL) are shown; *NS*, not significant; *, *p* < 0.05; **, *p* < 0.005. *C*, flow cytometry analysis of HIF reporter cells cultured for 24 h in FBS, LPDS, or LPDS supplemented with 0.5 or 1.5 mg/ml human LDL. *D*, immunoblots of whole-cell lysates from HIF reporter cells cultured for 24 h in FBS, LPDS, or LPDS with the indicated concentrations of human LDL. CALNEXIN served as a loading control.

To determine the lipoprotein requirement for this signaling pathway, we tested the ability of different lipoproteins to suppress HIF activation in LPDS. As expected, the addition of a mixture of bovine lipoproteins prevented SREBP activation in LPDS, confirming the proper delivery of extracellular lipids to cells (Fig. 2*A*). Likewise, lipoprotein addition inhibited HIF1α and HIF2α accumulation in a dose-dependent fashion (Fig. 2*A*), demonstrating that HIFα accumulated under normoxia due to a reduction in the supply of extracellular lipoproteins. Very low-density lipoprotein (VLDL), low-density lipoprotein (LDL), and high-density lipoprotein (HDL) are major lipoprotein species in human serum. These lipoproteins differ in their composition of triglycerides, cholesterol, and glycerophospholipids (18). When added at serum concentrations, both LDL and VLDL, but not HDL, inhibited HIF1α accumulation (Fig. 2*B*), indicating that low-density lipoproteins signal to HIF in Pa03c cells. LDL was most effective at suppressing HIF activation. Consistent with this observation, flow cytometry and immunoblotting using the Pa03c-derived HIF reporter cells demonstrated that human LDL blocked HIF activation in a dose-dependent manner (Fig. 2, *C* and *D*). Thus, low-density lipoproteins suppress HIF activation under normoxia.

After endocytosis, lysosomes degrade LDL particles and release lipids to regulate cellular lipid homeostasis. Whereas LDL catabolism requires lysosomes, lipids also regulate lysosome function (19). Multiple studies demonstrate that disruption of lysosomal proton homeostasis in the presence of oxygen results in activation of HIF transcription factors through disruption of amino acid homeostasis (20, 21, 22). To investigate whether LPDS-induced HIF activation is similarly mediated by disruption of lysosomal proton homeostasis, we measured lysosomal pH upon lipoprotein depletion. Lysosomal pH in cultured cells is 4.5–5.0 (23). As a positive control, inhibition of the lysosomal H^+^-ATPase with bafilomycin A1 raised lysosomal pH from 4.9 to 8.6 (Fig. S2). In contrast, lysosomal pH was unchanged in cells cultured in LPDS compared with FBS, indicating that lipoprotein regulation of HIF is not due to disruption of lysosomal proton homeostasis. In summary, these rescue experiments indicate that serum LDL regulates HIFα under normoxia.

### Lipoproteins regulate HIFα stability by controlling HIFα prolyl hydroxylation

**Figure 3:**
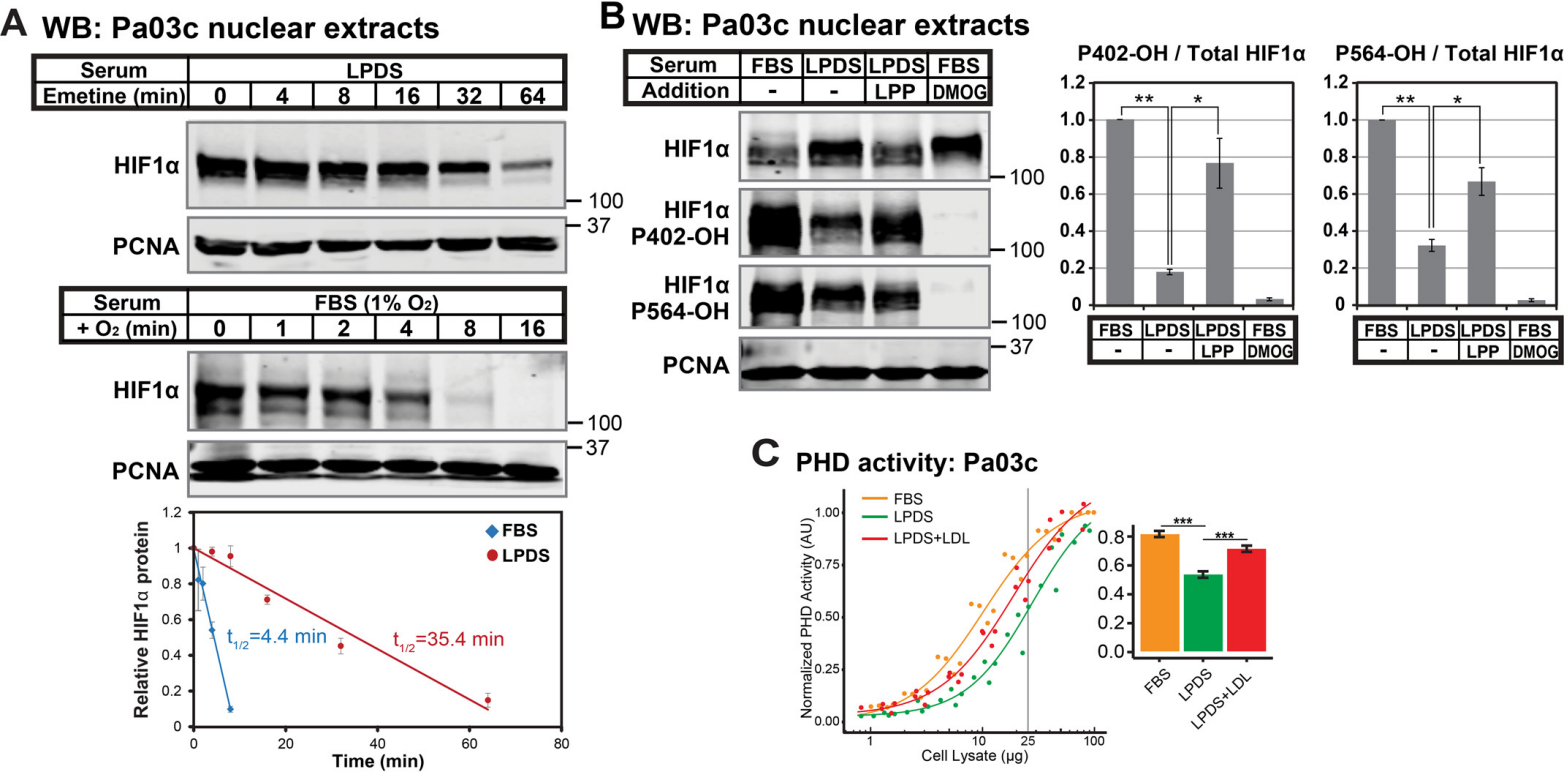
**Lipoproteins regulate HIFα stability by controlling HIFα prolyl hydroxylation.***A*, immunoblots (*WB*) of nuclear extracts from Pa03c cells cultured in LPDS for 16 h prior to treatment with the translation inhibitor emetine (25 μm) for the indicated time (*top*) or Pa03c cells cultured in FBS at 1% O_2_ for 4 h and then shifted to normoxia for the indicated time (*bottom*). HIF1α was normalized to PCNA signal and plotted relative to the *t* = 0 time point. Linear regression curves were used to calculate HIF1α *t*_½_ (*n* = 3, mean ± S.E. (*error bars*)). *B*, immunoblots of nuclear extracts from Pa03c cells cultured for 14 h in FBS or LPDS with the following additions: bovine lipoproteins (*LPP*; 1 mg/ml) or the PHD inhibitor DMOG (1 mm), followed by the addition of MG132 (10 μm) to all conditions for an additional 2 h. HIF1α signal was normalized to PCNA, and the level of hydroxylated HIF1α relative to total was normalized to that in FBS (*n* = 3, mean ± S.E.). *p* values from a single-column *t* test (LPDS *versus* FBS) or Student's *t* test (paired, LPDS + LPP *versus* LPDS) are shown; *, *p* < 0.05; **, *p* < 0.005. *C*, PHD activity assay of cell lysates from Pa03c cells cultured in FBS, LPDS, or LPDS with human low-density lipoprotein (1 mg/ml) for 16 h. Four-parameter log logistic models were fit to data obtained from at least three independent experiments. A bar plot shows the calculated PHD activities from the curves at 25 µg. ***, *p* < 0.0005; Student's *t* test.

To understand how serum lipoproteins repress HIFα accumulation in the presence of oxygen, we first examined gene expression of HIF subunits and other pathway regulators. mRNA expression of these genes was either unchanged or, in the case of the negative regulator *PHD2*, increased (Fig. S3, A and B), suggesting that lipoproteins do not regulate HIFα through changes in transcription. Oxygen regulates HIFα stability by controlling activity of PHD prolyl hydroxylases, which hydroxylate two proline residues on HIFα, enabling ubiquitination by the VHL-containing E3 ligase and subsequent proteasomal degradation (1). Both HIF1α and HIF2α are regulated by prolyl hydroxylation. For our mechanistic studies, we focused on HIF1α due to the availability of high-quality, commercial reagents. To investigate whether lipoproteins regulate HIF1α protein stability, we conducted chase experiments to measure the *t*_½_ of HIF1α. The *t*_½_ of HIF1α in the presence of oxygen has been calculated to be less than 5 min (24). Consistent with this previous study, HIF1α was rapidly degraded in the presence of oxygen in Pa03c cells (*t*_½_ = 4 min, Fig. 3*A*). In response to lipoprotein depletion, the *t*_½_ of HIF1α increased to 35 min, demonstrating that lipoproteins control HIF1α degradation. Mechanistically, lipoprotein depletion blocked HIF1α Pro-402 and Pro-564 hydroxylation, and lipoprotein addition rescued HIF1α prolyl hydroxylation (Fig. 3*B*). We hypothesized that lipoproteins promote PHD enzyme activity, resulting in increased prolyl hydroxylation on HIF1α and subsequent degradation. To test this idea, we developed an *in vitro* assay to directly measure PHD enzyme activity (Fig. S3, C and D). Lipoprotein depletion reduced PHD enzyme activity in cell lysates, and LDL addition rescued PHD enzyme activity (Fig. 3*C*). Taken together, we conclude that lipoprotein depletion inhibits PHD activity, leading to decreased prolyl hydroxylation on residues 402 and 564 and subsequent HIF1α accumulation.

### LDL regulation of HIFα requires mitochondria

PHD-mediated prolyl hydroxylation of HIFα requires molecular oxygen, and hypoxic PHD inactivation results from the limiting availability of oxygen (25). Alternatively, under other stress conditions, mitochondria-derived reactive oxygen species (ROS) can inhibit PHD enzymes and activate HIFα, and this response requires intact electron transfer in Complex III (26, 27). This signaling pathway is independent from hypoxia, which regulates HIFα in a mitochondrial ROS-independent manner (25). Because oxygen was not limiting in our experiments, we hypothesized that HIF up-regulation in LPDS was due to the inactivation of PHD enzymes by mitochondrial ROS.

**Figure 4:**
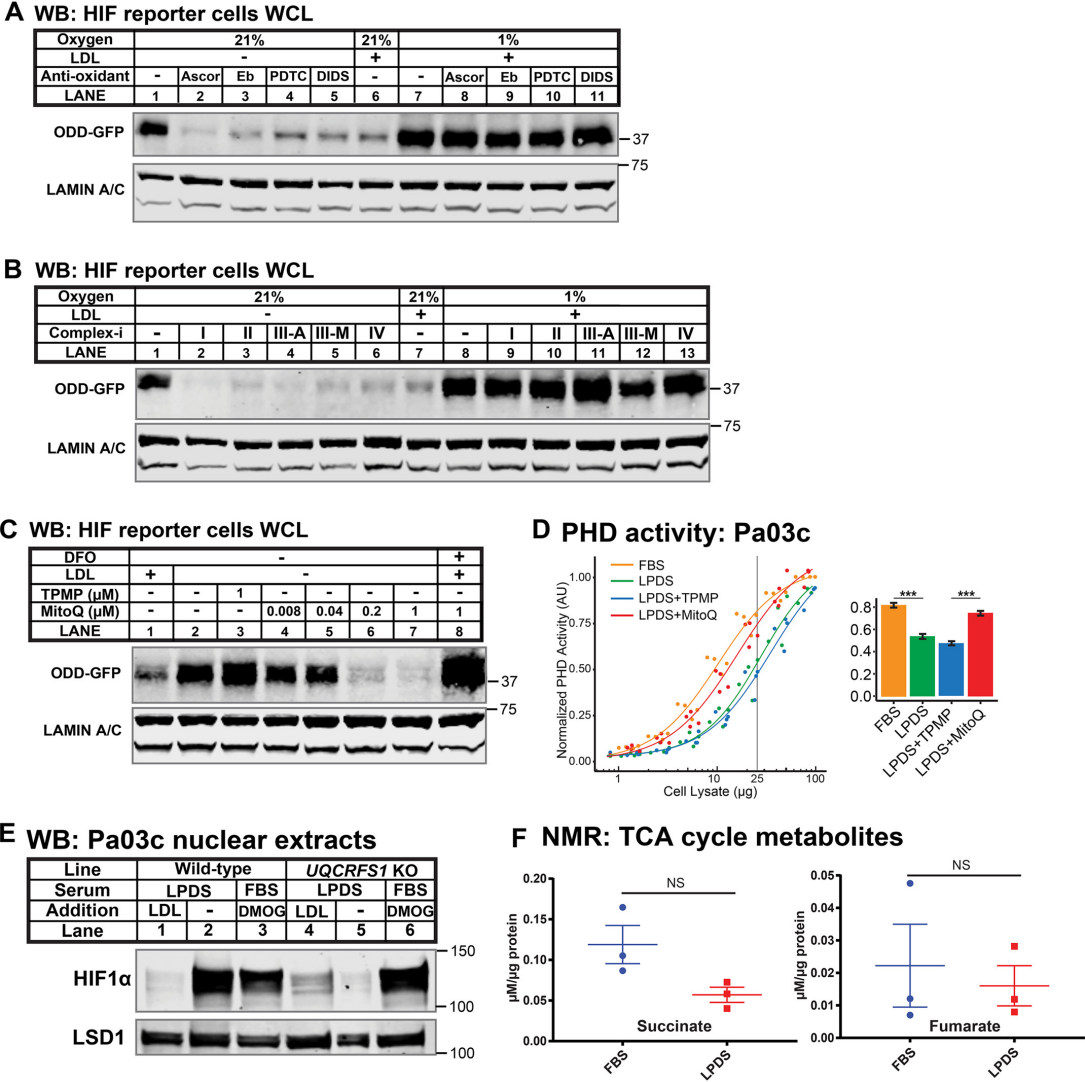
**LDL regulation of HIFα requires mitochondria.***A*, immunoblots (*WB*) of whole-cell lysates from HIF reporter cells cultured for 24 h in LPDS, LPDS with LDL (1 mg/ml), or LPDS with LDL (1 mg/ml) at 1% O_2_ with the indicated antioxidants: ascorbate (*Ascor*; 25 μm), ebselen (*Eb*; 25 μm), PDTC (20 μm), or 4,4′-diisothiocyanostilbene-2,2′-disulfonic acid (*DIDS*; 15 μm). LAMIN A/C served as a loading control. *B*, immunoblots of whole-cell lysates from HIF reporter cells cultured for 24 h in LPDS, LPDS with LDL (1 mg/ml), or LPDS with LDL (1 mg/ml) at 1% O_2_ with the indicated mitochondrial complex inhibitors (*Complex-i*): I (rotenone, 2 μm), II (malonate, 5 mm), III-A (antimycin, 10 μm), III-M (myxothiazol, 1 μm), or IV (oligomycin, 2 μm). LAMIN A/C serves as a loading control. *C*, immunoblots of whole-cell lysates from HIF reporter cells cultured for 24 h in LPDS with LDL (1 mg/ml); LPDS with mitochondrial-targeted chemical TPMP (1 μm) or antioxidant MitoQ (0.008–1 μm); or LPDS with LDL (1 mg/ml) plus the iron chelator (DFO; 100 μm) and mitochondrial-targeted antioxidant MitoQ (1 μm). LAMIN A/C served as a loading control. *D*, PHD activity assay of cell lysates from Pa03c cells cultured for 16 h in FBS, LPDS, or LPDS with mitochondrial-targeted chemical TPMP (1 μm) or antioxidant MitoQ (1 μm). Four-parameter log logistic models were fit to data obtained from at least three independent experiments. The bar plot shows the calculated PHD activities from the curves at 25 µg. ***, *p* < 0.0005, Student's *t* test. *E*, immunoblots of nuclear extracts from Pa03c cells or *UQCRFS1* KO Pa03c cells cultured for 16 h in LPDS with LDL (1 mg/ml) or LPDS or FBS with DMOG (1 μm). LSD1 served as a loading control. *F*, succinate and fumarate levels in cell extracts measured by NMR from Pa03c cells cultured in FBS or LPDS for 16 h. *NS*, *p* > 0.05, Student's *t* test. *Error bars*, S.E.

Consistent with a requirement for ROS, the addition of different antioxidants prevented accumulation of the HIF reporter ODD-GFP in LPDS, whereas HIF reporter activation under 1% oxygen was unaffected (Fig. 4*A*). The addition of chemical inhibitors against the different respiratory complexes prevented HIF reporter activation in LPDS, but not under 1% oxygen (Fig. 4*B*), demonstrating that HIF activation required an intact mitochondrial respiratory chain. Given this mitochondrial requirement, we next tested whether signaling could be repressed by mitoubiquinone (MitoQ), a mitochondria-targeted antioxidant (28). MitoQ consists of a mitochondria-targeting moiety triphenylmethylphosphonium (TPMP) and the antioxidant ubiquinone. MitoQ treatment blocked LPDS-induced HIF reporter activation in a dose-dependent manner, whereas TPMP had no effect (Fig. 4*C*, *lanes 3–7*). MitoQ suppression of the HIF reporter was not due to indirect effects on HIF reporter expression, because treatment with the iron chelator deferoxamine (DFO), which directly inhibits PHD enzymes, robustly activated the HIF reporter in the presence of MitoQ (Fig. 4*C*, *lane 8*). To test whether mitochondrial ROS acts by inhibiting PHDs, we assayed PHD activity *in vitro* under parallel conditions. As observed previously (Fig. 3*C*), lipoprotein depletion decreased PhD activity (Fig. 4*D*). Treatment with MitoQ restored PHD activity to levels in lipid-rich FBS, whereas TPMP treatment had no effect, indicating that mitochondrial ROS inhibited PHD activity in LPDS.

Either knocking out *UQCRFS1* (encoding Rieske Fe-S protein) or depletion of mitochondrial DNA (ρ^0^ cells) disrupts electron transport in Complex III (26, 27). Consistent with the requirement of the respiratory chain for LPDS-induced HIF activation (Fig. 4*B*), deletion of *UQCRFS1* and loss of mitochondrial DNA both blocked HIF activation in LPDS (Fig. 4*E* and Fig. S4 (A and B)). Elevated levels of succinate or fumarate in the mitochondria can inhibit PHD enzymes and increase HIFα in a ROS-independent manner (29, 30). To investigate whether lipoproteins signaled to HIF through a similar mechanism, we measured succinate and fumarate levels in cells. Lipoprotein depletion did not increase either succinate or fumarate (Fig. 4*F*), indicating that HIF activation did not result from increases in these TCA cycle metabolites. Taken together, we conclude that lipoprotein depletion activates HIFα by inhibition of PHD activity through a pathway that requires an intact mitochondrial respiratory chain and is inhibited by antioxidants.

### LDL-derived fatty acids regulate HIFα

**Figure 5:**
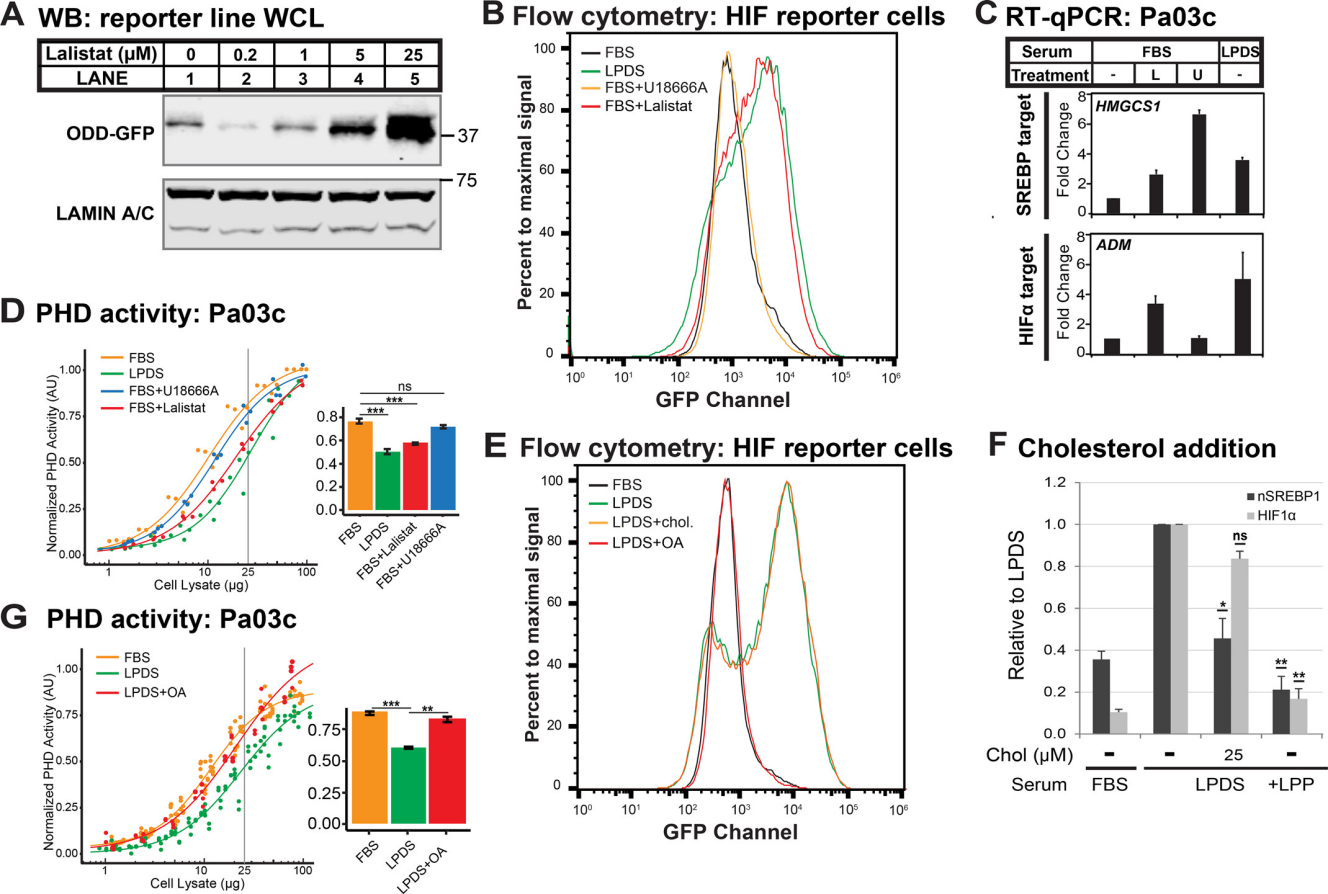
**LDL-derived fatty acids regulate HIFα.***A*, immunoblots (*WB*) of whole-cell lysates from HIF reporter cells cultured for 24 h in FBS with the indicated concentrations of lalistat 2, a specific LAL inhibitor. LAMIN A/C served as a loading control. *B*, flow cytometry analysis from HIF reporter cells cultured for 24 h in FBS, LPDS, or FBS with lalistat (25 μm) or U18666A (2 μm). *C*, Pa03c cells were cultured for 16 h in FBS, LPDS, or FBS with lalistat (25 μm) or U18666A (2 μm). Gene expression for SREBP or HIF transcriptional targets measured by RT-qPCR was normalized to vehicle-treated Pa03c cells cultured in FBS. *Error bars*, S.E. of -fold changes from three biological replicates (mean ± S.E.). *D*, PHD activity assay of cell lysates from Pa03c cells cultured in FBS, LPDS, or FBS with lalistat (25 μm) or U18666A (2 μm) for 16 h. Four-parameter log logistic models were fit to data obtained from at least three independent experiments. The bar plot shows the calculated PHD activities from the curves at 25 µg. ***, *p* < 0.0005, Student's *t* test. *E*, flow cytometry analysis from HIF reporter cells cultured for 24 h in FBS, LPDS, or LPDS supplemented with methyl-β-cyclodextrin complexed cholesterol (*chol*., 25 μm) or albumin-conjugated oleic acid (*OA*; 800 μm). *F*, immunoblots of nuclear extracts from WT Pa03c cells (*n* = 3) cultured for 16 h in FBS or LPDS in the absence or presence of water-soluble methyl-β-cyclodextrin-cholesterol complex (*M*β*CD-chol*) or in the presence of bovine lipoproteins (100 mg/dl). *p* values from a single-column *t* test (LPDS with MβCD-chol *versus* LPDS) are shown. *ns*, not significant; *, *p* < 0.05; **, *p* < 0.005. *G*, PHD activity assay of cell lysates from Pa03c cells cultured in FBS, in LPDS with fatty acid–free albumin (0.75%), or in fatty acid–free albumin–conjugated oleic acid (800 μm) for 16 h. Four-parameter log logistic models were fit to data obtained from at least three independent experiments. The bar plot shows the calculated PHD activities from the curves at 25 µg. ***, *p* < 0.0005; **, *p* < 0.005, Student's *t* test.

Next, we sought to understand what molecular component of LDL suppresses HIF activation. After endocytosis, LDL is transported to lysosomes where lysosomal acid lipase (LAL) hydrolyzes cholesteryl esters and triglycerides to release cholesterol and fatty acids (31). Lalistat is a specific chemical inhibitor of LAL (32, 33). Treatment of cells cultured in the presence of lipoprotein with the LAL inhibitor lalistat activated the HIF reporter ODD-GFP in a dose-dependent manner (Fig. 5*A*) (32), suggesting that an LDL-derived lipid signals to HIF. To cross-lysosomal membranes, cholesterol requires the Niemann–Pick C1 transport protein, whose activity is inhibited by the cationic sterol U18666A (34). In the presence of lipoproteins, lalistat induced HIF reporter expression to the same extent as lipoprotein depletion (Fig. 5*B*), and LAL inhibition increased expression of the HIFα target *ADM* and the SREBP target gene *HMGCS1* in Pa03c cells (Fig. 5*C*). Under the same conditions, inhibition of lysosomal cholesterol export with U18666A potently induced SREBP target gene expression (Fig. 5*C*) but failed to activate the HIF reporter (Fig. 5*B*), suggesting that LDL-derived cholesterol does not regulate HIF. Consistent with this, lalistat treatment, but not U18666A treatment, decreased PHD enzyme activity in the presence of lipoproteins (Fig. 5*D*).

Oleic acid is the most abundant fatty acid in human lipoproteins (35). To test directly whether LDL-derived fatty acids or cholesterol signal to HIF, we treated HIF reporter cells cultured in LPDS with either oleate conjugated to albumin or cholesterol complexed with methyl-β-cyclodextrin. Oleate addition completely suppressed activation of the HIF reporter, whereas cholesterol addition had no effect (Fig. 5*E*). Cholesterol addition blocked LPDS-induced SREBP activation, but not HIF activation (Fig. 5*F*), demonstrating that cholesterol was efficiently delivered to cells but failed to regulate HIF. Finally, oleate addition restored PHD enzyme activity in the absence of lipoproteins (Fig. 5*G*). In mitochondria, fatty acids can serve as a carbon source for the production of cellular ATP through β-oxidation. Inhibition of mitochondrial fatty acid oxidation by etomoxir, a small-molecule inhibitor of carnitine palmitoyltransferase-1, did not activate the HIF reporter (Fig. S5), indicating that fatty acids signal to HIFs independently of their role in energy production. Collectively, we conclude that LDL-derived fatty acids regulate HIFα under normoxia.

### Lipids regulate HIFα in animals

**Figure 6:**
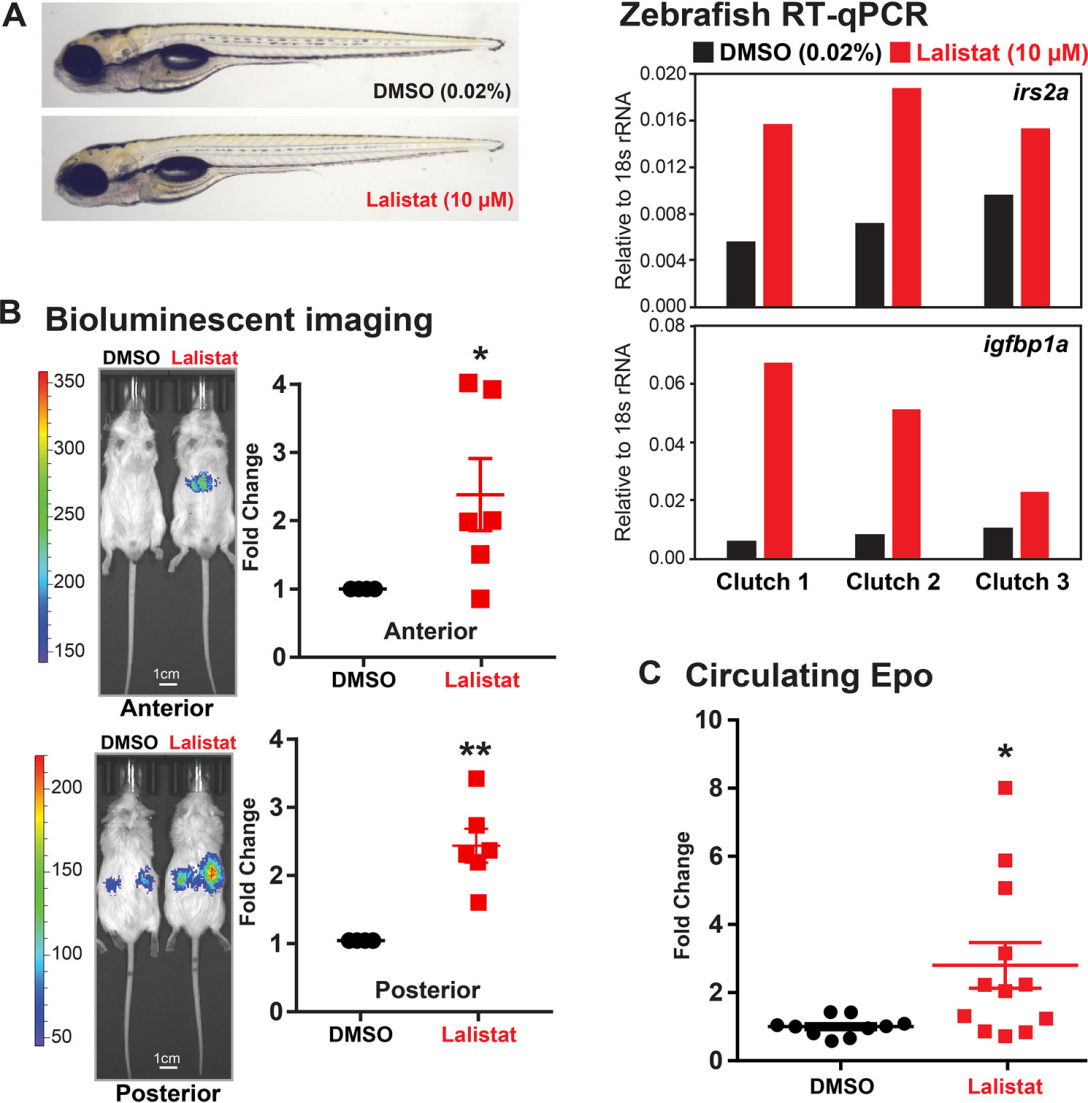
**LAL inhibition activates HIFα in animals.***A*, representative images of WT zebrafish larvae (5 days postfertilization) treated with lalistat (10 μm) or vehicle control DMSO (0.02%) for 2 h are shown. Expression of HIFα targets *irs2a* and *igfbp1a* (relative to *18s rRNA*, RNA pooled from five larvae) was analyzed using RT-qPCR. *B*, ODD-Luc mice received subcutaneous injection of either DMSO or lalistat (20 mg/kg) three times per week for 2 weeks. Shown are representative bioluminescent images (captured 2 min after luciferin injection) of ODD-Luc mice treated as indicated. Bioluminescent signals from lalistat-treated mice were normalized to vehicle control (*n* = 6, mean ± S.E. (*error bars*)). *p* values from a single-column *t* test (Lalistat *versus* DMSO) are shown: *, *p* < 0.05; **, *p* < 0.005. *C*, circulating Epo levels from lalistat-treated mice (*n* = 12, mean ± S.E.) were normalized to vehicle control (*n* = 10, mean ± S.E.). *p* values from a single-column *t* test (lalistat *versus* DMSO) are shown: *, *p* < 0.05.

To investigate whether lipids regulate HIFα in animals, we examined HIF signaling both during development in zebrafish larvae and in adult mice. Given our cultured cell results (Fig. 5), we employed lalistat as a tool to modulate lipoprotein-derived fatty acid supply to cells *in vivo*. Unbiased genome-wide transcriptional profiling analysis of zebrafish larvae treated with lalistat for 24 h returned “response to hypoxia” as the top enriched GO-term, and 9 of 24 up-regulated genes were known HIF targets in zebrafish (Fig. S6A) (36). Indeed, even acute lalistat treatment for 2 h robustly induced HIF target gene expression (Fig. 6*A* and Fig. S6 (B and C)). In line with our cell experiments, treatment of zebrafish larvae with the lysosomal cholesterol export inhibitor U18666A activated SREBP target genes, but not HIF target genes (Fig. S6D). Next, we examined fatty acid signaling to HIF in adult animals. The HIFα ODD-Luc reporter mouse ubiquitously expresses a bioluminescent reporter consisting of firefly luciferase fused to the HIF1α oxygen degradation domain, which confers PHD-dependent regulation on luciferase activity. In published experiments, administration of a clinical PHD inhibitor increased luciferase activity, which was readily visualized in mouse liver and kidneys (37). Similarly, lalistat administration increased ODD-luciferase activity both in mouse liver (Fig. 6*B*, *anterior view*) and kidneys (Fig. 6*B*, *posterior view*). HIF controls erythropoietin expression in mouse kidney in response to hypoxia (38). Consistent with activation of endogenous HIF, lalistat treatment increased serum erythropoietin (Fig. 6*C*), indicating that lalistat reduced PHD enzyme activity *in vivo*. Thus, we conclude that limiting lipid supply by LAL inhibition can regulate HIF both during development and in adult animals.

## Discussion

Oxygen and lipids play essential roles in cell function. Given the importance of these molecules, it is not surprising that mechanisms exist to communicate between the pathways controlling their supply. Oxygen supply impacts lipid metabolism and homeostasis through multiple mechanisms. Molecular oxygen is required for cholesterol synthesis at four different reactions, and the stearoyl-CoA desaturase enzyme that produces oleate requires oxygen. Thus, lipid synthesis is directly tied to oxygen supply. Further, oxygen regulates activity of HMG-CoA reductase in isoprenoid and cholesterol synthesis by controlling the enzyme's stability (39). Under hypoxia, HIF induces expression of Insig2, which accelerates the degradation of HMG-CoA reductase and down-regulates isoprenoid synthesis. Insig2 also regulates SREBP transcription factors, but it is unknown whether HIF regulation of Insig2 also affects SREBP activity in mammalian cells. Finally, HIF-dependent gene expression regulates lipid homeostasis by controlling lipid uptake, storage, and catabolism through a variety of mechanisms (11, 12, 13, 14). These examples highlight ways in which oxygen and HIF control lipid homeostasis, but little is known about how lipids in turn might regulate HIF.

**Figure 7:**
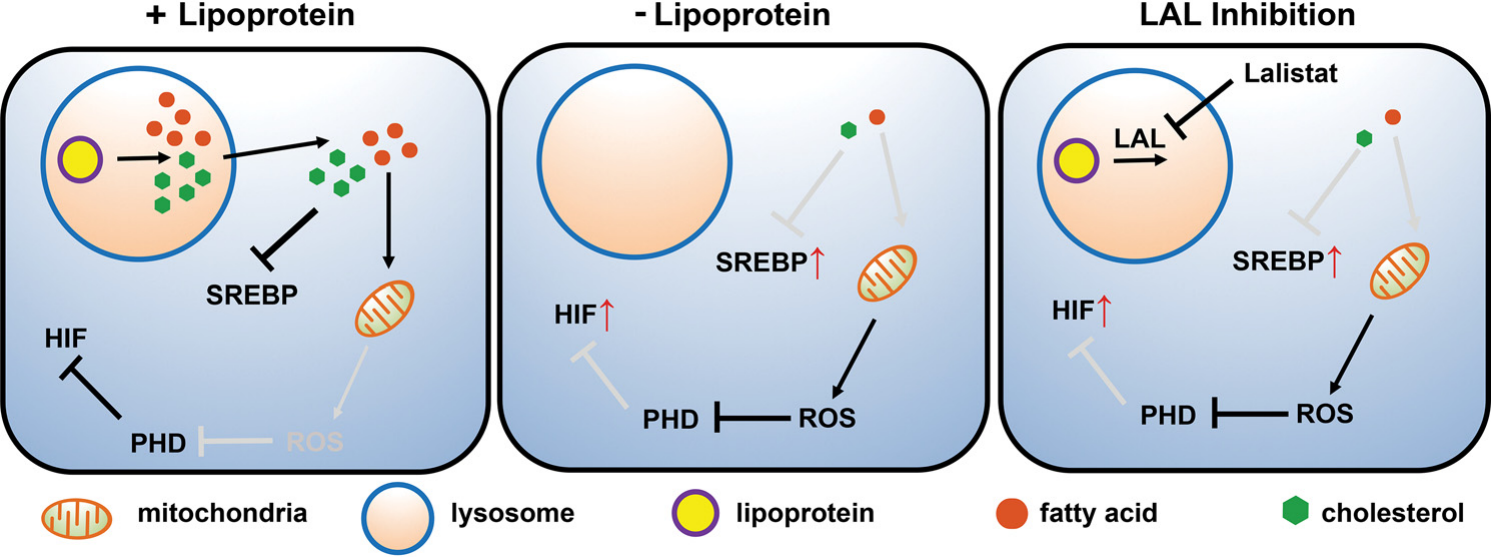
**Model for lipoprotein regulation of HIF and SREBP.***Left*, lipoproteins are directed to lysosomes through receptor-mediated endocytosis, where free cholesterol and fatty acids are released by LAL-catalyzed hydrolysis. Released cholesterol blocks SREBP activation, and fatty acids prevent ROS production from mitochondria. PHD remains active and hydroxylates prolyl residues on HIFα, which leads to its rapid degradation. *Middle*, in the absence of lipoproteins, cells are deprived of cholesterol and fatty acids, resulting in SREBP activation and mitochondrial stress. Mitochondrial-ROS production increases, thereby inactivating PHD and stabilizing HIFα. *Right*, upon LAL inhibition, cholesterol and fatty acids from cholesteryl esters and triglycerides are unavailable. Decreased cholesterol supply activates SREBP, and decreased fatty acid supply results in mitochondrial stress. As with lipoprotein depletion, stressed mitochondria produce ROS, which inactivates PHD and stabilizes HIFα. Organelles are not to scale.

Here, we report the discovery of a signaling pathway between lipoprotein-derived fatty acids and HIF, the master regulator of oxygen homeostasis. Our data support the model outlined in Fig. 7. When cultured in the presence of lipoproteins, HIFα subunits are hydroxylated by PHD enzymes, ubiquitinylated, and degraded, thereby repressing activity of the heterodimeric HIF transcription factor. In the absence of lipoproteins, cells are depleted for the unsaturated fatty acid oleate, which leads to generation of mitochondrial ROS that inhibits PHD enzymes and up-regulates HIF. Low oxygen signals to HIF by decreasing a substrate for the PHDs. Whereas fatty acids also signal by controlling PHD activity, this is independent of oxygen signaling because fatty acid depletion increases HIF levels in the presence of oxygen. Many details of this new signaling pathway remain to be elucidated. However, its physiological importance is underscored by the fact that inhibiting the release of fatty acids from the lysosome activated HIF in two different animal models (Fig. 6).

Cells acquire fatty acids from three sources: lipoproteins in the form of cholesteryl esters and triglycerides, free fatty acids bound to serum albumin, and *de novo* synthesis. Our studies indicate that in cultured cancer cells, lipoproteins are an important source of unsaturated fatty acids required for normal cell function because lipoprotein depletion disrupted cellular homeostasis, leading to HIF activation. Human LDL repressed HIF signaling most efficiently in a pancreatic cancer cell line (Fig. 2*B*). VLDL also repressed HIF signaling, whereas HDL had little effect. This differential lipoprotein activity may be due to differences in lipid composition of the particles or cell-specific differences in lipoprotein receptor expression. Indeed, other lipoproteins or nonalbumin-bound extracellular lipids may also signal to HIF. A detailed understanding of the lipoprotein and lipid signaling requirements for this pathway will require further experiments.

Experiments using the LAL inhibitor lalistat demonstrated that lipoprotein-derived fatty acids are required to repress HIF when cells are cultured in the presence of lipoproteins. Cholesterol is transported from lysosomes through a well-described pathway that requires the cholesterol-binding proteins NPC2 and NPC1 (40). Chemical inhibition of NPC1 interrupted the supply of free cholesterol, as indicated by activation of SREBP, but had no effect on HIF (Fig. 5). Furthermore, delivery of cholesterol to cells failed to repress HIF in the absence of lipoproteins, suggesting that cholesterol does not signal to HIF. It is possible that cholesterol may signal to HIF in some settings but that levels of cholesterol synthesis in cells examined in this study are sufficient to support mitochondrial homeostasis in the absence of lipoproteins. How lipoprotein-derived fatty acids exit the lysosome is not well-understood. Indeed, an efficient mechanism must exist as hydrolysis of each cholesteryl ester molecule generates one molecule of cholesterol and one molecule of fatty acid. Future studies of this signaling pathway may reveal genes required for fatty acid transport.

In our studies, blocking lipoprotein-derived fatty acid supply inhibited PHD enzymes by a mitochondria-dependent pathway, preventing HIFα degradation. How mitochondria sense changes in fatty acid supply to regulate HIF is unknown at this point. Oleic acid is the most abundant fatty acid in serum (35), and the addition of oleic acid blocked LPDS-induced HIF activation (Fig. 5, *E* and *G*). Fatty acids vary in length of carbon chain and number of double bonds. It will be interesting to test whether fatty acids other than oleic acid, namely saturated fatty acids, polyunsaturated fatty acids, or short-chain/long-chain fatty acids, prevent HIF activation upon lipoprotein depletion. In addition to glucose, most tissues also utilize fatty acids as fuel, to generate ATP through β-oxidation in the mitochondria. Etomoxir, a carnitine palmitoyltransferase 1a inhibitor, blocks fatty acid oxidation by preventing the import of fatty acid into the mitochondria. Interestingly, etomoxir failed to activate HIF activation in the presence of lipoproteins (Fig. S5), indicating that fatty acids modulate mitochondrial function independently from β-oxidation. Fatty acids are also structural components of phospholipids, which are essential for proper membrane and organelle function. A decreased supply of oleate could perturb mitochondrial membrane homeostasis, leading to mitochondrial dysfunction. Alternatively, changes in membrane homeostasis could directly impact the function of nonmitochondrial membranes, which in turn indirectly impacts mitochondrial function. For example, alterations in plasma membrane function could lead to defects in nutrient uptake that in turn impact mitochondrial function. Future studies will map where fatty acids act to influence mitochondrial function.

Lipoprotein depletion decreased HIF1α prolyl hydroxylation, resulting in accumulation of HIF1α (Fig. 3*B*). Mechanistically, accumulation of HIF1α required mitochondrial DNA and a functional respiratory chain (Fig. 4). Lipoprotein depletion or inhibition of lysosomal fatty acid supply inhibited PHD enzyme activity *in vitro* (Figs. 3*C* and 5*D*), and the addition of lipoprotein, oleate, or the mitochondrial antioxidant mitoQ restored PHD enzyme activity (Figs. 3*C*, 4*D*, and 5*G*). PHD enzymes require reduced ferrous iron, molecular oxygen, and 2-oxoglutarate sodium salt (2OG) for hydroxylation activity. Hypoxia directly inactivates PHDs by limiting oxygen availability, and the regulation is independent of ROS or mitochondrial respiration (25) (Fig. 4, *A* and B). However, our cultured cell experiments and *in vitro* PHD activity assays were conducted at 21% oxygen, so an independent mechanism must regulate PHD activity in response to lipoprotein depletion. Accumulation of the TCA cycle intermediates succinate and fumarate can inhibit PHD activity in cells (29, 30), but these metabolites were unchanged or decreased upon lipoprotein depletion (Fig. 4*F*). Collectively, these data suggest that lipoprotein depletion leads to respiratory chain defects and generation of reactive species (*e.g.* ROS), which in turn inhibit PHD activity.

Our *in vitro* PHD activity assay recapitulated the lipid regulation of PHD activity observed in intact cells. The *in vitro* assay contains saturating amounts of 2OG and oxygen, indicating that factors other than substrate availability inhibit PHD activity. Data indicate that ROS can inactivate PHDs through oxidation of ferrous iron to ferric iron (27, 41); however, the PHD assay also included ascorbate to prevent the oxidation of ferrous iron and increase enzyme turnover (42). Accumulating evidence shows that post-translational modification of PHDs under oxidative condition results in enzyme inhibition (43, 44). Under lipoprotein depletion, PHD modification could result in protein damage and conformational changes. Alternatively, binding to the iron co-factor could be decreased. Whereas the *in vitro* assay data point to post-translational regulation of PHDs, additional mechanisms to inhibit PHD activity, such as iron oxidation, may be at play in intact cells. Considering the distinct mechanisms on HIF activation by lipid depletion and hypoxia, these two HIF signaling pathways likely operate in parallel and are integrated at the level of PHD activity. Understanding exactly how PHD enzyme activity is regulated by fatty acid supply will require further detailed studies.

Collectively, our studies reveal a new signaling pathway whereby serum lipoproteins regulate activity of HIF. As mentioned above, oxygen regulates lipid metabolism through HIF-dependent regulation of HMG-CoA reductase c and unsaturated fatty acid synthesis (39, 45). But why should fatty acids control HIF activity? Under hypoxia, HIFs transcriptionally activate glucose transporters, pyruvate dehydrogenase kinase, and lactate dehydrogenase to shift glucose metabolism from oxidative phosphorylation to glycolysis (46, 47). In parallel, HIFs induce *BNIP3* and *BNIP3L* to promote mitochondrial-selective autophagy (48). These processes allow cells to adapt to hypoxic conditions by maintaining energy production and reducing hypoxia-induced mitochondrial ROS release when oxygen supply is low (3). Accumulating evidence also shows that hypoxia regulates lipid uptake (*FABP3/7*, *VLDLR*), storage (*PLIN2*, *ADFP*), and catabolism (*MCAD*, *LCAD*) in an HIF-dependent manner, so HIF may be activated to restore homeostasis (10, 11, 12, 13, 14). HIF is increasingly recognized as part of an antioxidant response that diverts pyruvate from the TCA cycle toward lactate production as a mechanism for reducing mitochondrial-derived ROS (49). As discussed above, respiratory chain–derived ROS may mediate the signal from mitochondria to PHDs, indicating mitochondrial stress under lipoprotein depletion. HIF activation may serve to down-regulate mitochondrial function as part of a cellular stress response.

The function of this signaling pathway in animals remains to be determined. In addition to cultured cells, we observed lipoprotein regulation of HIF in both developing and adult animals (Fig. 6), demonstrating its physiological relevance. *In vivo*, tissues acquire oxygen and lipoprotein from blood, and these two inputs may collaborate to regulate HIF-dependent production of blood vessels and red blood cells through activation of erythropoietin and VEGF, respectively. HIF activation is a common feature of poorly vascularized, solid tumors. To date, HIF activation has been attributed to low oxygen. However, tumors are similarly depleted of other nutrients; one of which could be lipoproteins. Although speculative, a lack of oleate supply could contribute to HIF activation in solid tumors. If true, these studies would have direct implications for the effect of dietary fatty acids on tumor metabolism. In conclusion, this study reveals that cellular lipid supply regulates HIF activity in an entirely new and unappreciated fashion, necessitating an examination of the contributions of lipid in control of HIF in normal physiology and disease.

## Experimental procedures

### Materials

We obtained reagents from the following manufacturers with catalog numbers in parentheses: RNA-STAT 60 from Tel-Test, Inc.; RNase-free DNase I (10104159001) and 1× cOmplete protease inhibitor without EDTA (11873580001) from Roche Applied Science; blocker casein in PBS (37528) and B-PER bacterial protein extraction reagent (78243) from Thermo; random primer mix (S1330), M-MuLV reverse transcriptase (M0253L), murine RNase inhibitor (M0314L), and Gibson Assembly Master Mix (E2611L) from New England Biolabs; GoTaq real-time PCR mix (A6002) from Promega; fetal bovine serum (FBS) (F2442, lot 15C376), lipoprotein-deficient serum (LPDS) (S5394, lot SLBQ5608V, prepared from fetal bovine serum F2442, lot 15C376), bovine lipoproteins (L4646), cholesterol (C8503), cholesterol/methyl-β-cyclodextrin complex (C4951), BSA/fatty acid–free (A8806), puromycin dihydrochloride (P8833), emetine dihydrochloride hydrate (E2375), ammonium pyrrolidine dithiocarbamate (PDTC, P8765), *N*-acetyl-l-cysteine (A9165), uridine (U3750), ascorbic acid (A5960), DFO (D9533), ethidium bromide (E7637), TPMP (468002; control for MitoQ), mevalonolactone (M4667; for sodium mevalonate preparation), oleic acid–albumin (O3008), d-luciferin synthetic (L9504), lalistat (for mouse experiment; SML2053), 2OG (K1875), and Amicon Ultra-15 centrifugal filter unit (UFC901008) from Sigma–Aldrich; LPDS (BT-907) from Alfa Aesar; DMOG (D1070) from Frontier Scientific; fetal bovine serum, heat-inactivated (S11150H) from Atlanta Biologicals; cell culture medium high-glucose DMEM (10-013) and sodium pyruvate (25-000-CI) from Corning Cellgro; penicillin-streptomycin (15140122), pHrodo-green–Dextran (P35368), Alexa Fluor 568–Dextran (D22912), and intracellular pH calibration buffer kit (P35379) from Thermo Fisher Scientific; PolyFect transfection reagent (301107) from Qiagen; site-1 protease inhibitor PF-429242 from Shanghai API Chemicals (947303-87-9); human lipoprotein/low density (437644), human lipoprotein/high density (437641), human lipoprotein/very low density (437647), *N*-acetyl-leucinyl-leucinyl-norleucinal (ALLN; 208719) from Millipore; U18666A (1638) and lalistat (for cell culture and zebrafish experiments; 6099) from Tocris Biosciences; ebselen (70530), oleic acid (90260), linoleic acid (90150), palmitic acid (10006627), and stearic acid (10011298) from Cayman Chemical; Mitoquinone mesylate (MitoQ) (317102) from Fisher; 3,3′,5,5′-tetramethylbenzidine (TMB) ELISA peroxidase substrate (TMBE-100) from Rockland. The Maxisorp ELISA plate (423501) was from NUNC. Disposable PD-10 desalting columns (17-0851-01) were from GE Healthcare. Oligonucleotides were made by Integrated DNA Technologies. Other general chemicals were obtained from Sigma or Thermo Fisher Scientific.

### Antibodies

We used the following antibodies: mouse monoclonal anti-HIF1α (clone 54, BD Biosciences, 610959); rabbit polyclonal anti-hydroxy-HIF1α-Pro-402 (Millipore, 07-1585); rabbit monoclonal anti-hydroxy-HIF1α-Pro-564 (clone D43B5, 3434), rabbit monoclonal anti-ARNT (clone D28F3, 5537), rabbit monoclonal anti-LSD1 (clone C69G12, 2184), and rabbit polyclonal anti-HDAC1 (2062) from Cell Signaling Technology; rabbit polyclonal anti-HIF2α (Novus Biologicals, NB100-122), mouse monoclonal anti-SREBP1 (clone 2A4, SC-13551), mouse monoclonal anti-Rieske FeS (RISP) (clone A5, SC-271609), and anti-PCNA (clone PC10, #SC-56) from Santa Cruz Biotechnology, Inc.; rabbit anti-thioredoxin (T0803) from Sigma; IRDye 800CW– or IRDye 680RD–conjugated goat anti-mouse or anti-rabbit secondary IgG from LI-COR; Alexa 594–conjugated goat anti-mouse (A11005) IgG from Invitrogen; and peroxidase affiniPure goat anti-rabbit IgG (111-035-144) from Jackson ImmunoResearch Laboratories.

### Animals

For zebrafish experiment, all procedures were approved by the Carnegie Institution Animal Care and Use Committee. WT (AB background) embryos were collected from natural spawning and raised in zebrafish embryo medium (50). The standard length of zebrafish larvae was measured from snout to caudal peduncle (51). For mouse experiments, all procedures were approved by the Institutional Animal Care and Use Committee at Johns Hopkins University School of Medicine. ODD-Luc bioluminescent reporter mice (FVB.129S6-*Gt*(*ROSA)26Sor^tm2(HIF1A/luc)Kael^*/J) expressing Hif-1α oxygen-dependent degradation domain fused to luciferase were obtained from the Jackson Laboratory (006206) (37). Mice were housed in a controlled environment with a 14-h light/10-h dark cycle and constant temperature (23 °C) and had free access to food (Teklad, 2018SX) and water. Homozygous male mice (∼7 weeks old) were used for all experiments. Over a 2-week treatment, lalistat or control solvent DMSO, was administrated subcutaneously at 20 mg/kg body weight three times a week, for a total of six injections (52). On the day following the final lalistat injection, all mice underwent IVIS imaging, followed by blood and tissue harvesting.

### In vivo luciferase activity assay

Mice were given 50 mg/kg body weight d-luciferin intraperitoneally under isoflurane-induced deep anesthesia. Mice were placed in a light-tight chamber of a Xenogen IVIS Spectrum Optical Imaging Device equipped with a photon-collecting camera (IS1651N7095, Andor, iKon). Live images were taken 2 min after d-luciferin injection at a fixed exposure time (0.5 s) for all studies and analyzed using Living Image software (IVIS Spectrum series 4.5.2.18424).

### Erythropoietin ELISA

Whole blood from the ODD-Luc bioluminescent reporter mice treated with Lalistat or vehicle control as described above was collected via cardiac puncture under deep anesthesia induced by inhalation of isoflurane into a 1.5-ml Eppendorf tube. Samples were kept on ice for 60 min and then spun at 2,000 rpm for 30 min at 4 °C. Serum was transferred to a new tube for erythropoietin analysis using a commercial ELISA kit specific for mouse erythropoietin (R&D Systems) according to the manufacturer's instructions.

### Cell culture

Cells were maintained in monolayer culture at 37 °C in 5% CO_2_. Pa03c is a human pancreatic cancer cell line that was generously provided by Dr. Anirban Maitra (Sidney Kimmel Comprehensive Cancer Center, Johns Hopkins University) (53). HEK293, HepG2, U2OS, ACHN, and BxPC3 were obtained from the American Type Culture Collection and maintained according to the supplier's instructions. WSC155 is a Pa03c-derived *HIF1A HIF2A* double knockout line, WSC238 is a Pa03c-derived *UQCRFS1* knockout line, and WSC55 is a Pa03c-derived *SCAP* knockout line (54). WSC238 and mitochondrial DNA–deficient ρ^0^ cells were maintained in DMEM (containing 100 units/ml penicillin and 100 μg/ml streptomycin sulfate) supplemented with 2 mm sodium pyruvate and 50 μg/ml uridine. WSC55 cells were maintained in DMEM (containing 100 units/ml penicillin and 100 μg/ml streptomycin sulfate) supplemented with 10% FBS, 5 μg/ml cholesterol, 1 mm sodium mevalonate, 20 μm sodium oleate. All other cells were maintained in DMEM (containing 100 units/ml penicillin and 100 μg/ml streptomycin sulfate) supplemented with 10% FBS. For experiments, cells were set up on day 0 at 3 × 10^6^ cells/100-mm dish (for Pa03c and derived lines) or 1 × 10^6^ cells/100-mm dish (for other cell lines) in maintaining medium. On day 1, cells were washed once by PBS and then refed with DMEM (containing 100 units/ml penicillin and 100 μg/ml streptomycin sulfate) supplemented with 10% FBS (Sigma F2442, lot 15C376) or matched LPDS (S5394, lot SLBQ5608V) for the indicated time. For hypoxic treatment, cells were incubated at 37 °C in a cell culture incubator (Series CB, BINDER) under 5% CO_2_ and different O_2_ concentrations.

### Preparation of BSA-conjugated oleic acid

The BSA-conjugated oleic acid preparation has been described previously (55). 45 mg of oleic acid were transferred to a glass beaker (50 ml) containing 1 ml of ethanol; 50 μl of NaOH (5 m) were added to the beaker to mix thoroughly. The ethanol was removed under nitrogen. The dried sodium oleate was solubilized in 5 ml of 150 mm NaCl and heated for 5 min at 60 °C. Then 6.25 ml of ice-cold 24% (w/v) bovine albumin (fatty acid–free) in 150 mm NaCl were added rapidly, and the clear solution was stirred for an additional 10 min. The final volume is adjusted to 12.5 ml with 150 mm NaCl and kept frozen at −20 °C until use. The final oleic acid concentration was 12.7 mm.

### Generation of knockout lines using CRISPR-Cas9 in Pa03c cells

Pa03c-derived *HIF1A HIF2A*-double knockout line WSC155 and *UQCRFS1* knockout line WSC238 were generated by CRISPR-Cas9–mediated genome editing. Human *HIF1A* gene (NM_001530.3) contains 15 exons and is translated into an 826-aa protein. A CRISPR guide RNA (gRNA) to target sequence 702–721 nucleotides (5′-GTTATGGTTCTCACAGATGA-3′) located in exon 3 (ENSE00003474252) was cloned into the Cas9-gRNA vector PX459 (Addgene 48139) (56). Human *HIF2A* gene (NM_001430.4) contains 16 exons and is translated into an 870-aa protein. A CRISPR gRNA to target antisense sequence 677–696 nucleotides (5′-GCTGATTGCCAGTCGCATGA-3′) located in exon 2 (ENSE00003556558) was cloned into the Cas9-gRNA vector PX459. Human Rieske iron-sulfur protein (RISP)-coding gene *UQCRFS1* (NM_006003.2) contains two exons and is translated into a 274-aa protein. A CRISPR gRNA to target antisense sequence 226–245 nucleotides (5′-AGGTCCAACACAGGCTGCTC-3′) located in exon 1 (ENSE00001124397) was cloned into the Cas9-gRNA vector PX459. To generate *HIF1A HIF2A*-double knockout line or *UQCRFS1* knockout line, Cas9-gRNA plasmids targeting both *HIF1A* and *HIF2A* or *UQCRFS1* were transfected into Pa03c cells using PolyFect transfection reagent (Qiagen). Transfected Pa03c cells were selected for growth in the presence of 1.5 μg/ml puromycin for 9 days. Single clones were isolated by dilution cloning. Genomic DNA flanking the gRNA target site was amplified by standard PCR and then sequenced by Sanger sequencing. Primer sequences are human *HIF1A* forward (5′-TAGCTTCTGGCCTGCACTTT-3′) and reverse 5′-CTTACCATTTCTGTGTGTA-AGC-3′), human *HIF2A* forward (5′-GGTTGTGTGTGGCTCAGACA-3′) and reverse (5′-GTGTTCTCCACAGCCTCTGG-3′), and human *UQCRFS1* forward (5′-GCAGGACTGCAGAAT-TTCCT-3′) and reverse (5′-CCAGCCCGACCTGATTCAGG-3′). One isolated clone, D11 (WSC155), contains a 1-bp deletion in *HIF1A* and both a 1-bp insertion and 7-bp deletion in *HIF2A* alleles. Knockout of *HIF1A HIF2A* was further confirmed by immunoblotting (Fig. S1D). One isolated clone G5 (WSC238) contains a 47-, 58-, and 88-bp deletion at the *UQCRFS1* locus, and the loss of UQCRFS1 was further validated by immunoblotting (Fig. S4A).

### Generation of mitochondrial DNA-deficient Pa03c cells

Mitochondrial DNA–deficient Pa03c ρ^0^ cells were generated using the ethidium bromide method (57). Briefly, Pa03c cells were selected in DMEM (containing 100 units/ml penicillin and 100 μg/ml streptomycin sulfate) supplemented with 50 ng/ml ethidium bromide, 2 mm sodium pyruvate, and 50 μg/ml uridine for 4 weeks. At the end of 4 weeks of exposure to ethidium bromide, cells were maintained in DMEM (containing 100 units/ml penicillin and 100 μg/ml streptomycin sulfate) supplemented with 2 mm sodium pyruvate and 50 μg/ml uridine. Total DNA was extracted using a Qiagen DNA blood minikit, and relative mitochondrial DNA copy number measurement was conducted using real-time PCR using primers targeting β_2_-microglobulin (forward, 5′-TGCTGTCTCC-ATGTTTGATGTATCT-3′; reverse, 5′-TCTCTGCTCCCC-ACCTCTAAGT-3′) and mitochondrially encoded tRNA leucine 1 (UUA/G) (forward, 5′-CACCCAAGAACAGGGTT-TGT-3′; reverse, 5′-TGGCCATGGGTATGTTGTTA-3′), respectively (58).

### Generation of HIF reporter line and flow cytometry

To construct the 5×HRE::ODD-GFP reporter plasmid, DNA sequence coding for human HIF1α_530–603_ was inserted 5′ of the GFP coding sequence in 5×HRE/GFP plasmid, which contains five copies of a 35-bp fragment from the hypoxia-responsive element (HRE) of the human *VEGF* gene and a human cytomegalovirus minimal promoter followed by GFP coding sequence (Addgene 46926) (59). The neomycin resistance gene downstream of the SV40 promoter was replaced by the hygromycin resistance gene. To generate Pa03c-derived 5×HRE::ODD-GFP reporter line WSC190, parental Pa03c cells were first infected by Cas9 lentivirus (Addgene 52962). Cells resistant to blasticidin (10 µg/ml) were transfected with 5×HRE::ODD-GFP plasmid and selected under 150 µg/ml hygromycin B. Clones resistant to both blasticidin and hygromycin B were isolated by dilution cloning. Clone WSC190 showed the greatest GFP induction upon iron chelator deferoxamine treatment. For flow cytometry analysis, Pa03c or WSC190 cells were seeded in a 6-well plate and treated under the indicated conditions for 24 h. Cells were trypsinized, resuspended in FACS buffer (1% FBS, 1 mm sodium EDTA, 25 mm HEPES, 155 mm NaCl, 1 mm KH_2_PO_4_, 3 mm Na_2_HPO_4_, pH 7.4), and assayed by an Attune NxT flow cytometer (Invitrogen). Data were analyzed and plotted using FlowJo software.

### Cell fractionation and immunoblotting

Mammalian cell fractionation has been described previously (60). Briefly, cultured cells (2–5 × 10^6^ cells) were allowed to swell in 0.5 ml of hypotonic buffer A (10 mm HEPES-KOH, pH 7.6, 10 mm KCl, 1.5 mm MgCl_2_, 1 mm sodium EDTA, 1 mm sodium EGTA, 250 mm sucrose, and a mixture of protease inhibitors: 5 μg/ml pepstatin A, 10 μg/ml leupeptin, 0.5 mm phenylmethylsulfonyl fluoride, 1 mm DTT, and 25 μg/ml ALLN) for 30 min on ice, quickly passed through a 22G1/2-gauge needle 30 times, and centrifuged at 890 × *g* at 4 °C for 5 min to pellet nuclei. The nuclear pellet was resuspended in 0.1 ml of buffer C (20 mm HEPES-KOH, pH 7.6, 0.42 m NaCl, 2.5% (v/v) glycerol, 1.5 mm MgCl_2_, 1 mm sodium EDTA, 1 mm sodium EGTA, and a mixture of protease inhibitors: 5 μg/ml pepstatin A, 10 μg/ml leupeptin, 0.5 mm phenylmethylsulfonyl fluoride, 1 mm DTT, and 25 μg/ml ALLN). The suspension was rotated at 4 °C for 1 h and centrifuged at 20,000 × *g* at 4 °C for 20 min. The supernatant was transferred to a new tube and designated as nuclear extract. Protein concentration in nuclear extracts was measured using the BCA Kit (Pierce), and samples were mixed with 5× SDS loading buffer (150 mm Tris-HCl, pH 6.8, 15% SDS, 25% (v/v) glycerol, 0.2% bromphenol blue, and 12.5% (v/v) β-mercaptoethanol) to a final concentration of 1×. After boiling at 100 °C for 5 min, protein samples (50 μg/lane) were separated by SDS-PAGE and transferred to nitrocellulose membranes using the Trans-Blot Turbo Transfer system (Bio-Rad), and membranes were incubated with primary antibodies indicated in the figure legends. Bound primary antibodies were visualized with IRDye 800CW or IRDye 680RD-conjugated goat anti-mouse or anti-rabbit IgG (working concentration: 0.2 μg/ml) using the LI-COR Odyssey CLx system according to the manufacturer's instruction. Working concentrations of primary antibodies were anti-HIF1α (0.25 μg/ml), anti-hydroxy-HIF1α-Pro-402 (1 μg/ml), anti-hydroxy-HIF1α-Pro-564 (1 μg/ml), anti-ARNT (1 μg/ml), anti-HIF2α (1 μg/ml), anti-PCNA (0.2 μg/ml), anti-HDAC1 (1 μg/ml), anti-RISP (0.2 μg/ml), anti-LSD (1:1,000), and anti-SREBP1 (5 μg/ml). Signal intensities of proteins were quantified by Image Studio software (LI-COR). To compare HIF1α levels between different conditions, normalized HIF1α signals were calculated by dividing signals from loading controls. -Fold change relative to the control condition was calculated by assigning HIF1α signal in the control as 1 and then performing a single-column *t* test.

### Gene expression analysis

Genome-scale gene expression analysis was conducted by the Sidney Kimmel Comprehensive Cancer Center at the Johns Hopkins Microarray Core Facility using Illumina HumanHT-12 bead arrays or the Agilent Zebrafish Gene Expression Microarray, respectively. RNA samples for microarray analysis were prepared using the Qiagen RNeasy RNA kit (human cells) or Zymo Direct-zol RNA kit (zebrafish larvae), respectively. In the microarray analysis of Pa03c cells, genes with a *p* value lower than 0.1 and signal higher than 100 in WT cells cultured in LPDS were selected for further analysis. Ninety-nine genes whose expression in Pa03c cells cultured in LPDS was up-regulated ≥2-fold compared with FBS were further analyzed for GO term enrichment using GOrilla (61). Redundant GO terms were trimmed using REVIGO (62), and GO terms with frequency higher than 2% were not shown (considered as not specific). The clustered heatmap containing all 99 up-regulated genes was generated using GenePattern 2.0 (63). In zebrafish microarray analysis, larvae (5 dpf) from three clutches were treated with vehicle (DMSO), U18666A (2 μm), or lalistat (25 μm), respectively (four animals in each arm) for 24 h. Differentially expressed probes were identified using the Limma package from Bioconductor (64). Data were loaded into the Shiny Volcano Plot website to generate the volcano plot (RRID:SCR_019194). RT-qPCR analysis of mRNA transcript abundance has been described previously (54). Briefly, total RNA (2 μg) was treated with RNase-free DNase I at room temperature for 15 min and followed by the addition of EDTA to a final concentration of 2.5 mm and incubation at 65 °C for 10 min. cDNA was synthesized using random primer mix and M-MuLV reverse transcriptase according to the manufacturer's instructions (New England Biolabs). cDNAs of the tested genes were quantified by real-time PCR using SYBR Green qPCR master mix. To compare expression of genes of interest under different conditions, the -fold change relative to control conditions was calculated with the ΔΔ*Ct* method using 36B4 (human cells) or 18S rRNA (zebafish) as the internal reference. The *p* value was calculated from three biological replicates using a single-column *t* test. Real-time PCR primer sequences are human *HIF1A* (forward, 5′-CTGGCTACAATACTGCACAAAC-3′; reverse, 5′-TGTGATCCAGCATTAAAGAACATAC-3′), human *HIF2A* (forward, 5′-CTTAGTCATGGTGT-TGCGTAAATC-3′; reverse, 5′-CGTCAGTAACCCTTCAAGTTCT-3′), human *ARNT* (forward, 5′-ACCCACA-ACCAGAGGAATCTA-3′; reverse, 5′-TGTCAGGATCAGGAGGACAA-3′), human *VHL* (forward, 5′-GGTGAAACC-TCATCTCCACTT-3′; reverse, 5′-GATTTCCTGACCTC-GTGATCC-3′), human *PHD1* (forward, 5′-CTGCTTCT-GACTTTGCCTCT-3′; reverse, 5′-CAAAGGTCTCTTC-TCCTCCTTG-3′), human *PHD2* (forward, 5′-ACTATCT-GTGGGTTGTGCTTG-3′; reverse, 5′-GGCCATCCTGAT-TTCTTGATCT-3′), human *PHD3* (forward, 5′-CACTGT-GGTTGGCAGTATGA-3′; reverse, 5′-CAGTGCGATC-TGTGCTTACT-3′), human *FIH1* (forward, 5′-GTGGT-TGCTGAAAGGGAAAC-3′; reverse, 5′-CCACTAGGTC-CTCTCATCTCT-3′), human *HMGCS1* (forward, 5′-GGTGTGCTCCTGAATCAGTTCATGGT-3′; reverse, 5′-AGGACTGCAACAACAAACTCCCTC-3′), human *ADM* (forward, 5′-TCGGACTCTGGTGTCTTCTAA-3′; reverse, 5′-GTACCATGGGCGCCTAAAT-3′), human *BNIP3* (forward, 5′-TGGATGCAGGTTGTCTACTAAAG-3′; reverse, 5′-AGCTGAGTTTGTAGCTCTATCTTG-3′), human *PFKFB4* (forward, 5′-CTGGAGGGTAGCACATCTTTC-3′; reverse, 5′-TAGCAGAGCAGCACACAAG-3′), zebrafish *18s rRNA* (forward, 5′-TGCAGAACCCTCGCCAGTACAAAATCCCAG-3′; reverse, 5′-CCAGAAGTGACGGAGACCACGGTGAGCCCT-3′), zebrafish *igfbp1a* (forward, 5′-CTTCTGAACTTCTTC-TGGGTGG-3′; reverse, 5′-CCCGTTATGAGACTCCGGATGAT-3′), and zebrafish *irs2a* (forward, 5′-ACACAGCTCT-GCCTCCGTAGA-3′; reverse, 5′-ACACAGCTCTGCCTCCGTAGA-3′).

### Indirect immunofluorescence microscopy

Pa03c cells were seeded on day 0 at a density of 5 × 10^4^ cells/well (6-well plate, 22 × 22-mm coverslip per well) in DMEM supplemented with 10% (v/v) FBS. On day 1, cells were washed with PBS and then incubated for 16 h under different culture conditions. Cells were processed for indirect immunofluorescence as described previously (65). Briefly, cells were fixed in freshly prepared 3% (w/v) paraformaldehyde in PBS at room temperature for 10 min and then permeabilized by 0.5% (v/v) Triton X-100/PBS (pH 7.4), 100 mm glycine for 3 min at room temperature. Primary antibodies (anti-HIF1α; 1:250) and secondary antibodies (Alexa-594 goat anti-mouse IgG; 1:250) in 1% BSA/PBS (pH 7.4), 100 mm glycine were incubated with coverslips at room temperature for 30 min each. Coverslips were washed three times with PBS (pH 7.4), 100 mm glycine after each incubation. Coverslips were then mounted to slides and dried in the dark overnight before visualization using a Zeiss AXIO Imager-M2 microscope. Images were captured by Zeiss Plan-Neofluar ×100/1.30 numerical aperture oil objective and processed using iVision software.

### In vitro PHD activity assay

The *in vitro* PHD activity assay was developed based on a similar plate-based assay (66). Pa03c cells were seeded (3 × 10^6^ cells/10-cm dish) on day 0. On day 1, cells were washed with PBS once and then refed with fresh medium with the indicated supplements for 17 h. Cells were trypsinized, washed once with PBS, and then resuspended in 1 ml of ice-cold hypotonic buffer (5 mm NaCl, 1.5 mm MgCl_2_, 20 mm HEPES-KOH, pH 7.5). One-tenth of cell suspensions (100 µl) were used for protein estimation by BCA assay. Remaining cells were centrifuged at 400 × *g* for 5 min at 4 °C, and the cell pellets were stored at −80 °C until lysis. Cell pellets were lysed in lysis buffer (5 mm KCl, 1.5 mm MgCl_2_, 1 mm 2OG, 1× cOmplete Protease Inhibitor without EDTA, 5 mm β-mercaptoethanol, 2 mm ascorbate, 1% (v/v) IGEPAL, 20 mm HEPES-KOH, pH 7.5). The lysates were centrifuged at 17,000 × *g* for 15 min at 4 °C, and then supernatants were used in the assay. Wells of Maxisorp ELISA plate were coated overnight with 0.5 µg of His_8_-GST-ODD1 in PBS (100 µl) at 4 °C and then blocked with casein for 1 h at room temperature. All subsequent procedures were conducted at room temperature. Cell lysates were serially 2-fold diluted, in duplicate, in 100 µl of reaction buffer (5 mm KCl, 1.5 mm MgCl_2_, 1 mm 2OG, 1×cOmplete Protease Inhibitor without EDTA, 5 mm β-mercaptoethanol, 2 mm ascorbate, 20 mm HEPES-KOH, pH 7.5), from 100 to ∼1.56 µg/well. Cell lysates were omitted from control wells. Cell lysates were incubated for 1 h, and then the wells were washed three times with 200 µl of wash buffer (100 mm NaCl, 10% (w/v) blocker casein, 0.05% (v/v) Tween 20, 20 mm Tris-HCl, pH 7.5). To detect hydroxylated ODD1, wells were incubated with 1.33 µg/ml VBC (67) in binding buffer (100 mm NaCl, 50 mm Tris-HCl, pH 7.5) for 15 min. Anti-thioredoxin primary antibody (1:8,000 in binding buffer) was added to each well containing the VBC solution for 30 min. Thereafter, goat anti-rabbit horseradish peroxidase–conjugated secondary antibody (1:400) was added to the wells for 30 min. After washing the wells three times with 200 µl of wash buffer, bound horseradish peroxidase was detected by the addition of 100 µl of TMB substrate for 5 min. The reaction was stopped by the addition of 100 µl of 1 m H_2_SO_4_, and absorbance at 450 nm was measured using a microplate reader (BMG Labtech). Specific absorbance was calculated by subtracting the no-lysate control readings from all the measurements of a particular treatment. The normalized PHD activities were determined by taking the ratio of the specific absorbance values from different treatments to the values of FBS control. A four-parameter log logistic dose-response curve (Hill equation) was fit to the results using the drc package in R (68). From the models, normalized PHD activity at 25 µg of cell lysate was predicted for each treatment, which was then represented as a bar plot with Student's *t* test significance values.

### Lysosomal pH measurement

Lysosomal pH was measured as described previously, with modifications (69). Briefly, Pa03c cells were seeded (6 × 10^5^ cells/6-well plate) on day 0. On day 1, cells were refed with fresh medium with pH-sensitive pHrodo-green–Dextran (5 μg/ml) together with pH-insensitive Alexa Fluor 568–Dextran (10 μg/ml) for 24 h, washed twice with PBS, and then chased in the indicated conditions for an additional 16 h. Cells for lysosomal pH calibration were trypsinized and then incubated in calibration buffer (pH 4.5, 5.5, 6.5, and 7.5) with 10 μm K^+^/H^+^ ionophore nigericin and 10 μm K^+^ ionophore valinomycin for 5 min at 37 °C. Treated sample cells were trypsinized and then resuspended in FACS buffer (1% FBS, 1 mm sodium EDTA, 25 mm HEPES, 155 mm NaCl, 1 mm KH_2_PO_4_, 3 mm Na_2_HPO_4_, pH 7.4). Cells were gated on a forward scatter and side scatter. pH-sensitive pHrodo Green signal (BL1) and pH-insensitive Alexa-568 signal (YL2) from single cells were obtained. A four-point linear pH calibration curve with different pH values (4.5, 5.5, 6.5, and 7.5) was generated using BL1/YL2 ratios from calibration controls, and lysosomal pH of treated samples was calculated using the calibration curve. *p* values were calculated from four biological replicates using one-way ANOVA.

## Data availability

Microarray data have been deposited in the GEO, with accession codes GSE129432, GSE129433, and GSE129434.
