# Supplementary material for: Serum lipoprotein–derived fatty acids regulate hypoxia-inducible factor
Source: J Biol Chem. 2021 Jan 13;295(52):18284–300. doi: 10.1074/jbc.RA120.015238 (PMC7939398; doi:10.1074/jbc.RA120.015238)
Supplement: Supplementary file 1 [file mmc1.pdf]

Supporting Information

Serum lipoprotein-derived fatty acids regulate hypoxia-inducible factor

Wei Shao, Jiwon Hwang, Chune Liu, Debaditya Mukhopadhyay, Shan Zhao, Meng-Chieh Shen, Ebru S. Selen Alpergin, Michael J. Wolfgang, Steven A. Farber, Peter J. Espenshade

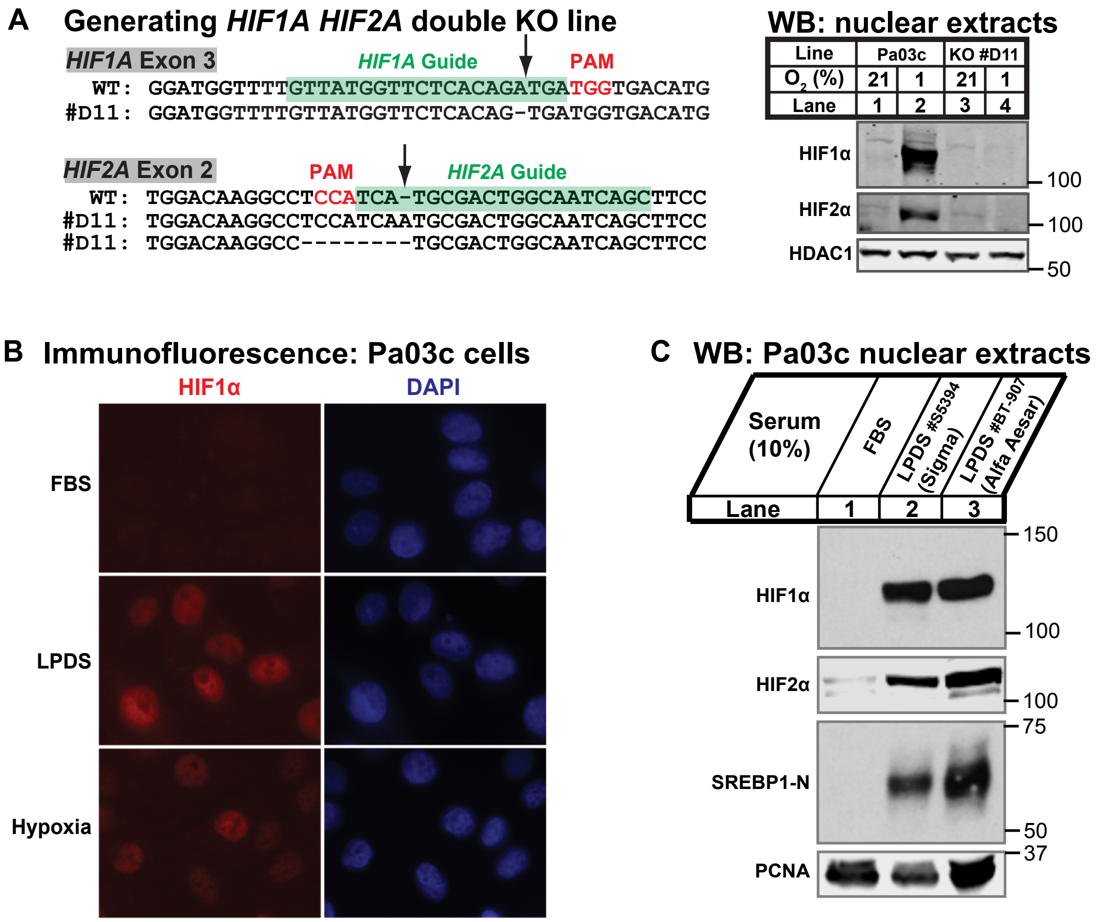

Fig. S1 Lipoprotein depletion activates HIFα under normoxia (related to Figure 1)
(A) A Pa03c-derived *HIF1A HIF2A* double knockout cell line (Clone #D11) was generated by CRISPR-Cas9 technology. Genomic DNA flanking the gRNA target site was amplified by standard PCR and sequenced by Sanger sequencing. Expected cleavage sites are indicated by arrows. Clone #D11 contains a 1 bp deletion in *HIF1A* and both a 1 bp insertion and a 7 bp deletion in *HIF2A*. Knockout of *HIF1A HIF2A* was further confirmed by immunoblotting nuclear extracts from cells cultured for 16 hours in DMEM supplemented with 10% (v/v) FBS under normoxic or hypoxic (1% O_2) conditions.
(B) Indirect immunofluorescence of Pa03c cells cultured for 16 hours in DMEM supplemented with 10% (v/v) FBS or LPDS under normoxic conditions, or in DMEM supplemented with 10% (v/v) FBS in 1% O_2 (Hypoxia). HIF1α was visualized by anti-HIF1α (1:250) followed by Alexa-594 labeled goat anti-mouse IgG (1:250). DAPI served as a nuclear staining control.
(C) To test whether the LPDS response was specific to a single commercial source, we compared serum from two different companies. Immunoblots of nuclear extracts from Pa03c cells cultured for 16 hours in DMEM supplemented with 10% (v/v) FBS or LPDS commercially available from indicated companies.

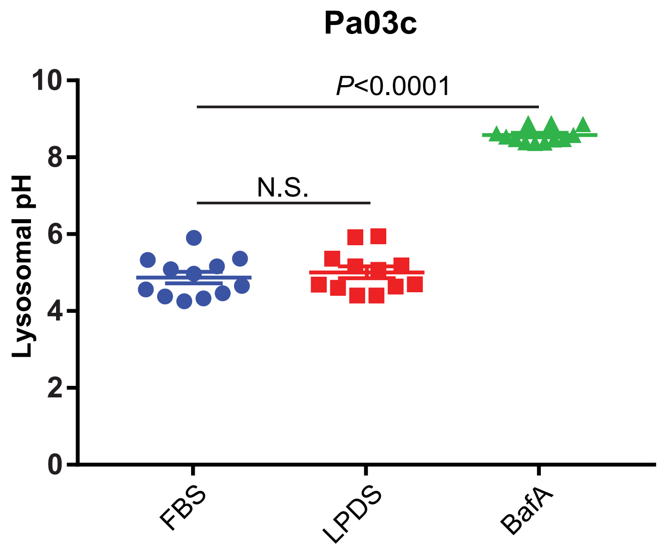

Fig. S2 Low-density lipoproteins regulate HIFα. (related to Figure 2)
Pa03c cells were loaded with pH-sensitive pHrodo Green/Dextran and pH-insensitive Alexa 568/Dextran for 24 hours, then chased in indicated condition for additional 16 hours. pH-sensitive pHrodo Green signal and pH-insensitive Alexa-568 signal from single cells were obtained using flow cytometry. A four-point pH calibration standard curve with different pH values (4.5, 5.5, 6.5 and 7.5) was generated in each experiment to calculate lysosomal pH values from indicated samples. Data (mean±SEM) from 12 replicates were plotted. *P* values were calculated using one-way ANOVA.

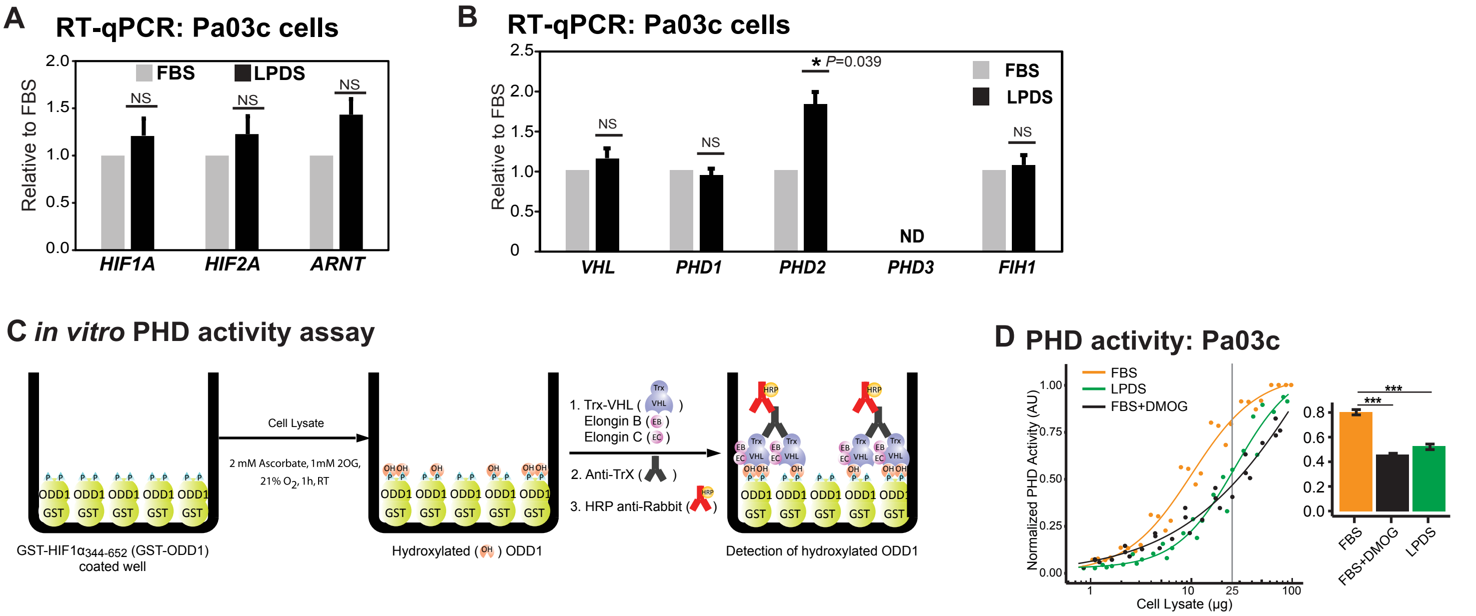

Fig. S3 Lipoproteins regulate HIFα stability by controlling HIFα prolyl hydroxylation (related to Figure 3)

(A, B) Pa03c cells were cultured for 16 hours in FBS or LPDS. Gene expression measured by RT-qPCR was normalized to cells cultured in FBS. Error bars represent the standard error of fold changes from 3 biological replicates (mean ± SEM). NS, not significant (P>0.05) from single column t-test (compared with FBS).

(C) Overview of the plate-based *in vitro* PHD activity assay: ELISA plates were coated with *E.coli*-expressed GST-HIF1α $ODD_{344\text{-}652}$. Cell lysates under various treatments were added and incubated at RT for 1 hour in the presence of ascorbate (2 mM) and 2-OG (2-oxoglutarate) (1 mM). PHD enzymes in cell lysates hydroxylated prolyl residues in substrates. Elongin B, C and pVHL (von Hippel –Lindau tumor suppressor, with thioredoxin tag, Trx-VHL) were then added and formed protein complexes specifically with hydroxylated substrates. Protein complexes were labeled by rabbit anti-thioredoxin antibody, followed with HRP-labeled secondary anti-rabbit IgG. HRP activity was then measured using TMB.

(D) Pa03c cell lysates from cells cultured in FBS, LPDS or FBS with a PHD inhibitor DMOG (1 mM) for 16 hours were analyzed using PHD activity assay. Four-parameter log logistic models were fit to data obtained from at least three independent experiments. Bar-plot shows the calculated PHD activities from the fitted curves at 25 μg. *P*<0.0005 (***), student's t-test.

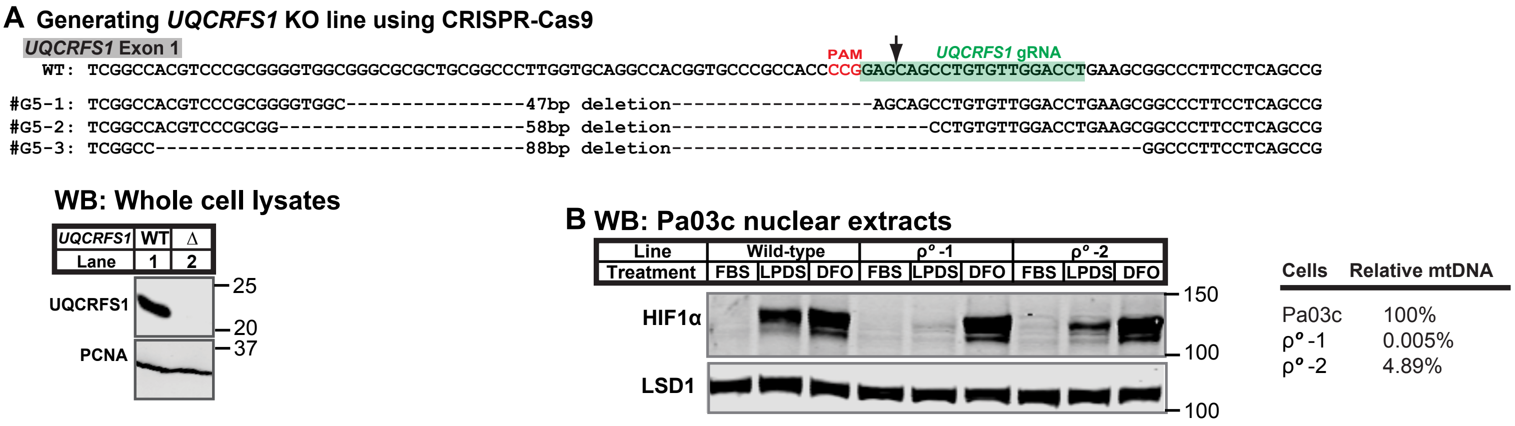

Fig. S4 LDL regulation of HIFα requires mitochondria (related to Figure 4).

(A) Pa03c-derived *UQCRFS1* knockout cell line was generated by CRISPR-Cas9 technology. Genomic DNA flanking the gRNA target site was amplified by standard PCR and sequenced by Sanger sequencing. Guide RNA sequences in front of the PAM (NGG) are highlighted, and expected cleavage sites are indicated by arrows. The clone #G5 contains 47 bp, 58 bp and 88 bp deletions in *UQCRFS1*. Knockout of *UQCRFS1* was further confirmed by immunoblotting.

(B) Immunoblots of nuclear extracts from Pa03c cells or mitochondrial DNA deficient ρ^0 cells cultured for 16 hours in FBS, LPDS or FBS with iron chelator (DFO, 100 μM). LSD1 serves as a loading control. Mitochondrial DNA abundance of two freshly-prepared ρ^0 pools was analyzed by real-time PCR.

WB: reporter line WCL

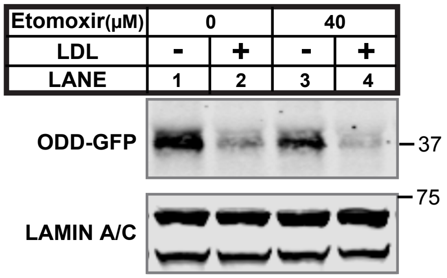

Fig. S5 LDL-derived fatty acids regulate HIFα. (related to Figure 5)
Immunoblots of whole cell lysates from HIF reporter cells cultured for 24 hours in LPDS or LPDS supplemented with LDL (1 mg/ml), with indicated concentrations of carnitine palmitoyltransferase-1 (CPT-1) inhibitor Etomoxir. LAMIN A/C serves as a loading control.

Fig. S6 LAL inhibition activates HIFα in animals (related to Figure 6).

A Zebrafish: Lalistat (25 µM, 24 hr)

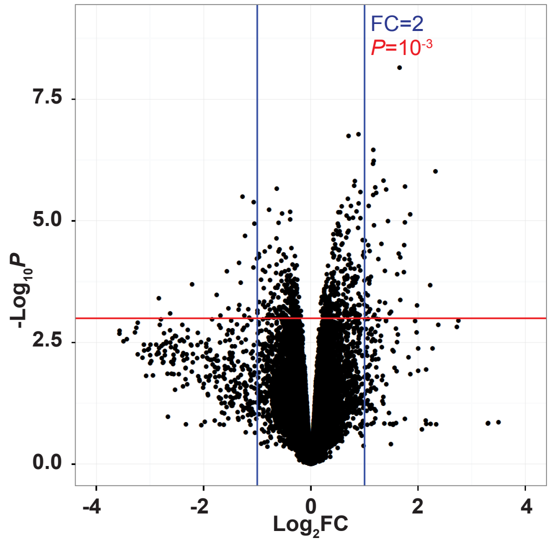

GO Term	Description	$log_{10}P$
**Up-regulated**		
GO:0001666	response to hypoxia	-5.375
GO:0042737	drug catabolic process	-3.133
GO:0014823	response to activity	-3.347
GO:0007259	JAK-STAT cascade	-4.041
GO:0032869	cellular response to insulin	-3.475
GO:0009628	STAT cascade	-4.041
**Down-regulated**		
GO:0097435	supramolecular fiber org.	-4.349
GO:0055002	striated muscle cell develop.	-4.261
GO:0031033	myosin filament organization	-4.010
GO:0007010	cytoskeleton organization	-3.454

B Zebrafish: Lalistat (10 µM, 2 hr)

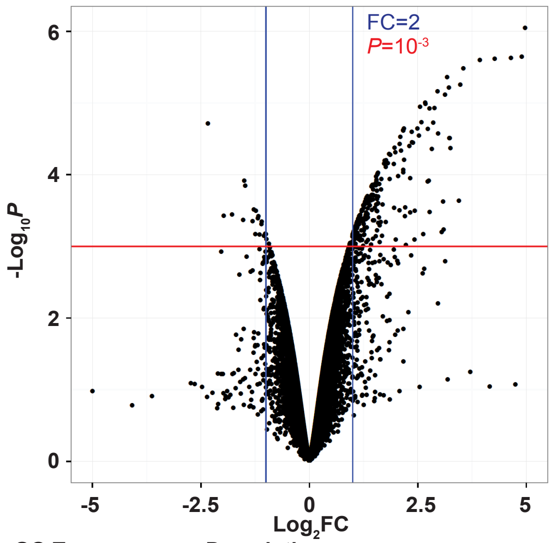

GO Term	Description	$log_{10}P$
**Up-regulated**		
GO:0015748	organophosphate ester transport	-3.932
GO:0032870	cellular response to hormone stimulus	-4.827
GO:1901568	fatty acid derivative metabolic process	-3.075
GO:0008285	negative regulation of cell proliferation	-4.342
GO:0001666	response to hypoxia	-3.400
GO:0018105	peptidyl-serine phosphorylation	-3.306
GO:0006094	gluconeogenesis	-3.907
GO:0032922	circadian regulation of gene expression	-3.657
GO:0033559	unsaturated fatty acid metabolic process	-3.452

Down-regulated
not identified

C Zebrafish size measurement

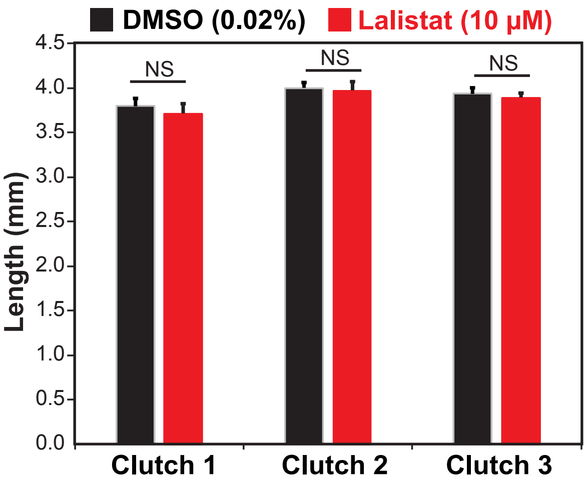

D Zebrafish: U18666A (2 µM, 24 hr)

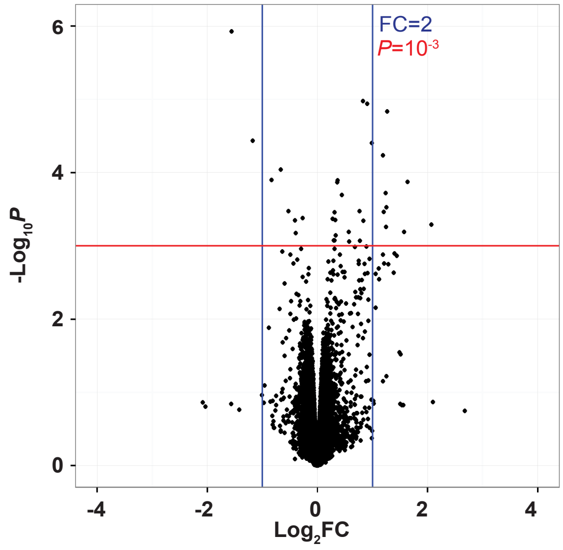

GO Term	Description	$log_{10}P$
**Up-regulated**		
GO:0008202	steroid metabolic process	-12.2741
GO:1901615	organic hydroxy comp. metabolic process	-10.4789
GO:0045337	farnesyl diphosphate biosynthetic process	-6.6364
GO:0016125	sterol metabolic process	-13.3382

Down-regulated
not identified

Fig. S6 LAL inhibition activates HIFα in animals (related to Figure 6)
(A) RNA samples from wild-type zebrafish larvae (5 dpf, 4 larvae for each treatment) treated with lalistat (25 μM) or vehicle control DMSO (0.1%) for 24 hours were analyzed by zebrafish gene expression microarray. Data from 3 biological replicates are presented in the volcano plot. The enriched GO terms were identified by GOrilla and REVIGO.
(B) RNA samples from wild-type zebrafish larvae (5 dpf, 4 larvae for each treatment) treated with lalistat (10 μM) or vehicle control DMSO (0.02%) for 2 hours were analyzed by zebrafish gene expression microarray. Data from 2 biological replicates were presented in the volcano plot. The enriched GO terms were identified by Gorilla and REVIGO.
(C) The standard length of three clutches of zebrafish larvae (5 dpf, 10 larvae for each treatment) treated with lalistat (10 μM) or vehicle control DMSO (0.02%) for 2 hours was measured. Error bars represent the standard error from 10 larvae (mean ± SEM). NS, not significant (P>0.05, student t-test).
(D) RNA samples from wild-type zebrafish larvae (5 dpf, 4 larvae for each treatment) treated with U18666A (2 μM) or vehicle control DMSO (0.1%) for 24 hours were analyzed by zebrafish gene expression microarray. Data from 2 biological replicates are presented in the volcano plot. The enriched GO terms were identified by Gorilla and REVIGO.
